# Synaptic changes contribute to persistent extra-motor behaviour deficits in amyotrophic lateral sclerosis

**DOI:** 10.1186/s40478-025-02150-5

**Published:** 2025-12-21

**Authors:** Wei Luan, Rebecca San Gil, Lidia Madrid San Martin, Maize C. Cao, Florencia Vassallu, Juliana Venturato, Phillip K. West, Heledd Brown-Wright, Adekunle T. Bademosi, Yi Jia Chye, Hao Yu Wu, Anna Harutyunyan, Katherine J. Robinson, Mu Sheen Chang, Catherine A. Blizzard, Emma L. Scotter, Lionel M. Igaz, Adam K. Walker

**Affiliations:** 1 Clem Jones Centre for Ageing Dementia Research, Queensland Brain Institute, The University of Queensland, St Lucia, QLD 4072 Australia; 2 School of Medical Sciences, Faculty of Medicine and Health, The University of Sydney, Camperdown, NSW 2050 Australia; 3 Charles Perkins Centre, The University of Sydney, Camperdown, NSW 2050 Australia; 4 School of Biological Sciences and Centre for Brain Research, University of Auckland, Auckland, 1010 New Zealand; 5 Grupo de Neurociencia de Sistemas, Departamento de Ciencias Fisiológicas, Facultad de Ciencias Médicas, Universidad de Buenos Aires, Buenos Aires, Argentina; 6 Instituto de Fisiología y Biofísica Bernardo Houssay (IFIBIO Houssay), CONICET - Universidad de Buenos Aires, Buenos Aires, Argentina; 7 Tasmanian School of Medicine, University of Tasmania, Hobart, TAS 7000 Australia; 8 Menzies Institute for Medical Research, University of Tasmania, Hobart, TAS 7000 Australia; 9 Sydney Pharmacy School, Faculty of Medicine and Health, The University of Sydney, Camperdown, NSW 2006 Australia

**Keywords:** TDP-43, ALS, Motor neuron disease, Frontotemporal dementia, Synapse, Extra-motor phenotypes, Proteomics, Transcriptomics

## Abstract

Extra-motor symptoms are increasingly recognised in amyotrophic lateral sclerosis (ALS), encompassing cognitive, social, and behavioural deficits. TAR DNA binding protein 43 (TDP-43) pathology is the central disease marker of almost all cases of ALS and approximately half of frontotemporal dementia (FTD). However, the mechanisms linking TDP-43 pathology with extra-motor symptoms in TDP-43-associated neurodegenerative diseases remain unresolved. In this study, we used the rNLS8 mouse model, which expresses human TDP-43 with an ablated nuclear localisation sequence (hTDP-43^∆NLS^) in a doxycycline-regulatable manner causing progressive motor decline reminiscent of ALS, to delineate molecular changes associated with disease-relevant phenotypes. We found that in addition to previously reported dramatic motor decline, rNLS8 mice also develop extra-motor phenotypes consistent with FTD, including disinhibition-like and anxiety-like behaviours, and social interaction impairments. These changes began in the earliest disease stages and remained readily detectable even when rNLS8 mice became severely motor impaired. Notably, extra-motor deficits persisted in rNLS8 mice that had recovered motor function upon hTDP-43^∆NLS^ transgene suppression. This correlates with widespread mis-splicing of RNA in rNLS8 cortex at disease onset with n = 814 genes showing differential exon usage, a molecular phenotype of TDP-43 loss of function. Mis-splicing persists in the rNLS8 cortex in recovery and may represent lasting impacts of cytoplasmic TDP-43 expression. Further, proteomics analysis of the cortex of rNLS8 mice revealed depletion of synaptic proteins, particularly those involved in glutamatergic signalling pathways, which also persisted following hTDP-43^∆NLS^ transgene suppression. Similar changes to the glutamatergic pathway were detected in transcriptomic and proteomic datasets from human ALS and FTD post-mortem cortex. Our findings suggest that targeting glutamatergic synaptic components may be an avenue to correct extra-motor deficits associated with TDP-43 pathology.

**Supplementary Information:**

The online version contains supplementary material available at 10.1186/s40478-025-02150-5.

## Introduction

Amyotrophic lateral sclerosis (ALS) is the most common motor neuron disease (MND), with relentlessly progressive decline due to neuromuscular degeneration, and average ~3-year lifespan after diagnosis [1]. In addition to the predominant motor manifestation, extra-motor changes, such as disinhibition and alterations in social and executive function, are now recognised as important features of ALS [2]. This appreciation of extra-motor components has resulted in a proposed revision of ALS diagnostic criteria to incorporate neuropsychological deficits [3, 4]. Indeed, up to 65% of people living with ALS develop extra-motor cognitive and behavioural symptoms [5], and up to 15% of people with ALS also meet the criteria for diagnosis of frontotemporal dementia (FTD) [6]. Notably, extra-motor symptoms adversely impact quality of life, resulting in cognitive, linguistic, mood, and social deficits, and are associated with shorter survival in people living with ALS [7]. Nevertheless, only ~ 1% of ALS research has focused on understanding these extra-motor symptoms [8] and less than 5% of ALS clinical trials have assessed neuropsychological symptoms as outcome measures [9]. Disease-modifying therapies for people living with FTD in the absence of ALS-associated motor impairments are also lacking. This suggests a need to better understand the mechanisms driving extra-motor manifestations in these diseases to inform future therapeutic avenues. Likewise, animal models recapitulating extra-motor features in the context of concomitant motor decline are required for preclinical assessment of relevance to the full experience of ALS [10].

Cytoplasmic mislocalisation and accumulation of TAR DNA-binding protein 43 (TDP-43) have been observed in up to 97% individuals with ALS and nearly 50% FTD cases [11]. Loss of TDP-43 function in the nucleus and accumulation of aggregated TDP-43 in the cytoplasm are concurrent events in neurons that are strongly linked to neurodegeneration [11]. However, the precise mechanisms by which TDP-43 pathology induces disease remain unclear. Evidence suggests that a large number of TDP-43 target genes are related to synaptic organisation and function [12, 13], disturbances of which appears to contribute to disease development and symptoms in ALS and FTD [14, 15]. Recent findings also highlight TDP-43-mediated RNA splicing and processing of critical axonal and synaptic components associated with neurodegeneration, for example, *STMN2* [16–18] and *UNC13A* [19–22]. It remains to be determined whether changes in levels of synaptic proteins are associated with extra-motor behavioural phenotypes in disease.

The ‘regulatable NLS’ (rNLS8) TDP-43 mouse model, which expresses human TDP-43 with a defective nuclear localisation sequence (NLS), hTDP-43^ΔNLS^, under doxycycline (dox)-suppressible promoter directed to neurofilament heavy chain (NEFH)-positive neurons, has become an established model for understanding the role of TDP-43 pathology in disease [23–27]. rNLS8 mice accumulate cytoplasmic insoluble TDP-43, and develop progressive motor neuron loss, dramatic motor dysfunction and decreased survival, which can be functionally restored by suppression of hTDP-43^ΔNLS^ expression [28]. In the rNLS8 mouse model, synaptic proteins are significantly decreased from 1 week off dox (WOD) [29], and significant neuronal loss is observed in cortical [28] and hippocampal [24] regions from 3–4 WOD, with later loss of spinal cord alpha motor neurons [28]. Notably, extra-motor behavioural deficits develop in transgenic mice with *CamkIIa* promoter brain-restricted hTDP-43^ΔNLS^ expression that lack typical ALS-like motor decline [30], and rNLS8 mice have been shown to develop features of hyperexcitability, hyperactivity and cognitive deficits [24, 25]. However, despite the known molecular, cellular, and motor deficits that recapitulate ALS in rNLS8 mice, the extra-motor phenotypes of this model have not been fully examined and the molecular mechanisms driving such features of disease are unknown, particularly during functional recovery.

In this study, we characterised extra-motor phenotypes and their molecular drivers in rNLS8 mice after short-term and long-term induction of neuronal hTDP-43^∆NLS^ expression. Firstly, we observed that short-term expression of hTDP-43^∆NLS^ led to persistent motor and extra-motor behavioural phenotypes in the rNLS8 mice that unexpectedly remained after motor recovery. Cortex transcriptomics showed that these behavioural phenotypes correlated with significant differential exon usage of neuronal genes, linking the dysregulation of splicing driven by TDP-43 RNA-binding with persistent extra-motor phenotypes. Furthermore, long-term induction of hTDP-43^∆NLS^ in rNLS8 mice caused persistent disinhibition-like behaviour, executive and habituation deficits, increased anxiety-like behaviour, and social impairment during and even after motor recovery. These behaviours correlated with a significant decrease in glutamatergic synapse components, suggesting that glutamatergic synapse deficits may underly extra-motor phenotypes. Further, the set of glutamatergic synapse proteins identified were also significantly decreased in published transcriptomic and proteomic datasets from human ALS and FTD post-mortem tissues. Overall, our data indicate that rNLS8 mice develop readily detectable extra-motor phenotypes independent of and despite motor decline, and display molecular signatures of TDP-43 loss-of-function and changes to synaptic protein levels. Importantly, not all behavioural and molecular phenotypes were readily reversible in this model, which is a relevant consideration for maintenance of quality of life for people with disease in a future with therapies that can effectively target TDP-43.

## Methods

### Animals

rNLS8 mice were produced from the intercross of  >10 generation back-crossed C57BL/6JAusb background homozygous *tetO*-hTDP-43^ΔNLS^ line 4 mice (B6.C3-Tg(tetO-TARDBP*)4Vle/JAusb, derived from Jackson Laboratories stock #014650) with hemizygous *NEFH*-tTA line 8 mice (B6.C3-Tg(NEFH-tTA)8Vle/JAusb, derived from Jackson stock #025397), constantly fed with dox-containing chow (200 mg/kg, Specialty Feeds, Australia) [28, 31]. The number of experimental mice per sex in individual tests can be seen in individual figure legends. Experiments were conducted with approval from the Animal Ethics Committee of The University of Queensland (#QBI/131/18 and 2022/AE000578). During experiments, male and female mice were switched to normal chow (SF00-100, Specialty Feeds, Australia) to induce expression of hTDP-43^∆NLS^ at approximately ten weeks of age. All mice were housed in temperature- and humidity-controlled conditions (21 ± 1 °C, 55 ± 5%) with a 12-h light/dark cycle (lights on at 06:00 h).

### Behaviour tests

#### Neurological score

Mice were assessed for observation of collapsing splay or clasping of hindlimbs on a scale of 0 to 4 by tail suspension for > 5 s as previously described [28].

#### Open field test

Open Field Assessment of general exploratory locomotion in a novel clear Plexiglas (30 cm × 30 cm × 50 cm) arena with white floor divided by two zones: periphery and centre (comprising 50% of the total area cantered) as previously described [30]. The activities were assessed during 30 min with illumination 30 LUX and analysed by time (bin = 5 min).

#### Social interaction test

Social interaction was assessed using a social approach test in a three chamber apparatus with identical Plexiglas chambers (40 × 40 × 40 cm) as previously described [32]. Before testing, the animal was allowed to explore freely for 5 min in the testing arena which served to reduce novelty-induced locomotor hyperactivity. During the sociability testing session, one wire cage contained an unfamiliar C57BL/6JAusb mouse (same sex as test mouse), and the other one contained a novel object made of black LEGO™ (Billund, Denmark). A camera above the maze captured videos. Social interaction time was analysed as nose orientation towards the wire cage within a 2-cm interaction zone adjacent to the wire cage which was outlined EthoVision analyses (Noldus, The Netherlands). During the social recognition session for assessment of social memory, the inanimate object was replaced by a new mouse as the ‘novel’ mouse, whereases, the previous ‘novel’ mouse became the ‘familiar’ mouse. The testing animal was placed in the centre of the middle chamber to explore for 5 min. Video records were defined and analysed as above by a trained experimenter who was blinded to the treatment.

#### Y-maze test

A Y-maze with three identical arms made of transparent Plexiglas (25 cm × 25 cm × 25 cm) placed at 120º angles to each other was used and placed in a room with clues to allow for visual orientation with illumination 30 lx as previously described [33]. Each mouse was placed at the end of one arm facing the centre and allowed to explore the maze freely for 8 min without training, or reward, while the experimenter remained out of sight. The percentage of spontaneous alternation was defined as the number of actual alternations divided by the possible alternations [(# alternations)/(total arm entries − 2) × 100]. Total entries were scored as an index of ambulatory activity in the Y maze and mice with scores below 12 were excluded as previously outlined [33].

### RNA extraction and analysis

#### RNA extraction

RNA was extracted with Qiazol (Qiagen #79,306) using the Qiagen RNeasy Mini Kit (Qiagen, #74,104) and Precellys tissue homogeniser (Bertin Instruments, Montigny-le-Bretonneux, France). On-column DNase I digestion was conducted using RNase-free DNase I (Qiagen #79,254). The concentration of extracted RNA was determined using a NanoRNA kit (Agilent #5067–1511) and Bioanalyzer (Agilent 2100, Santa Clara, CS, USA).

#### Transcriptomic analysis

RNA was purified from the right rostral cortex, hippocampus, and lumbar spinal cord of control and rNLS8 mice (*n* = 4/group, 2 male and 2 female) at disease onset (2 WOD) and recovery (2 WOD followed by 6 weeks on dox). The rostral cortex was chosen as it contains the frontal and temporal-parietal regions relevant to ALS and FTD. The quality of total RNA samples was determined by Agilent Bioanalyzer. Samples with RNA Integrity Number (RIN) > 8.0 were then used for library construction. RNA was analysed by RNA-seq (polyA enriched, strand-specific, Illumina paired-end 150 bp, 20 M reads) with Azenta Life Science. Quality assessment of the data was determined (FastQC, v0.10.1), low quality reads and adapter sequences were removed (Cutadapt, v1.9.1), and aligned (Hisat2, v2.0.1) to the reference genome (*Mus musculus*, ensembl, GRCm39.107). Transcriptomic data was analysed for differential gene expression (DESeq2 [34], v1.6.3) and differential exon usage (DEXSeq [34], v1.18.4) as a measure of alternative splicing.

#### POSTAR3 analysis of TDP-43 binding sites

Transcripts with DEU events identified in the disease onset (2 WOD) and recovery (2 WOD followed by 6 weeks on dox) were analysed for TDP-43 binding capacity using the publicly available CLIP-seq database accessible via POSTAR3 [35]. The CLIPdb module of POSTAR3 was used to identify known binding sites of human TDP-43 and mouse TDP-43 . POSTAR3 output was crossed referenced with onset and recovery transcript lists to calculate the proportion of transcripts known to bind with human or mouse TDP-43.

### Meta-analysis of proteomics data

To understand the molecular signatures of the cortex during recovery, we re-analysed a published longitudinal proteomics dataset of the rNLS8 cortex [29]. Briefly, all proteins that were significantly decreased in the rNLS8 cortex in late disease (6 WOD) were analysed for their protein abundance at recovery (6 WOD followed by 2 weeks on dox). These proteins were then grouped into subsets of proteins that demonstrated “recovered” (i.e., returned to control levels) or “persistent” (i.e., remained significantly decreased) signatures. The relative protein abundance of each protein was presented using the ComplexHeatmap [36] package in RStudio.

To align our findings in the rNLS8 mouse cortex with datasets from human post-mortem brain tissue, we used TDP-map (https://shiny.rcc.uq.edu.au/TDP-map/) [29]. Briefly, with the dataset choice set to “human”, the protein subset of interest was input into the IDs field (including *TARDBP*/TDP-43 for comparison) and log fold change plots and pie chart plots were saved from the respective tabs.

### Gene ontology analysis

Protein or gene subsets were deposited into Metascape [37] or QIAGEN Ingenuity Pathway Analysis (v 01.21.03) [38] for analysis of enriched biological terms and identification of protein–protein interaction networks.

### Statistical analysis

For data from two groups, statistical analyses were conducted using a two-tailed t-test after confirming normality assumptions. For data from four groups, Two-way ANOVA was used for analyses of datasets with Bonferroni’s post hoc test using Prism-GraphPad software (version 9). Statistical significance is indicated as *p < 0.05, **p < 0.01, and ***p < 0.001. Data are presented as mean ± standard error of the mean (SEM) or ± standard deviation (SD) as shown in the figure legends.

### Data availability statement

The transcriptomic data has been deposited to the Sequence Research Archive (PRJNA1298393). Previously published mouse proteomics [29], human proteomics [39] and human transcriptomics [40] datasets used in this work for comparative analyses are available from the respective references. Supplementary Data are provided with this paper and any additional information required to re-analyse the data reported in this paper is available from the corresponding authors upon request.

## Results

### Short-term TDP-43 cytoplasmic accumulation induces persistent hyperlocomotion in rNLS8 mice

**Fig. 1:**
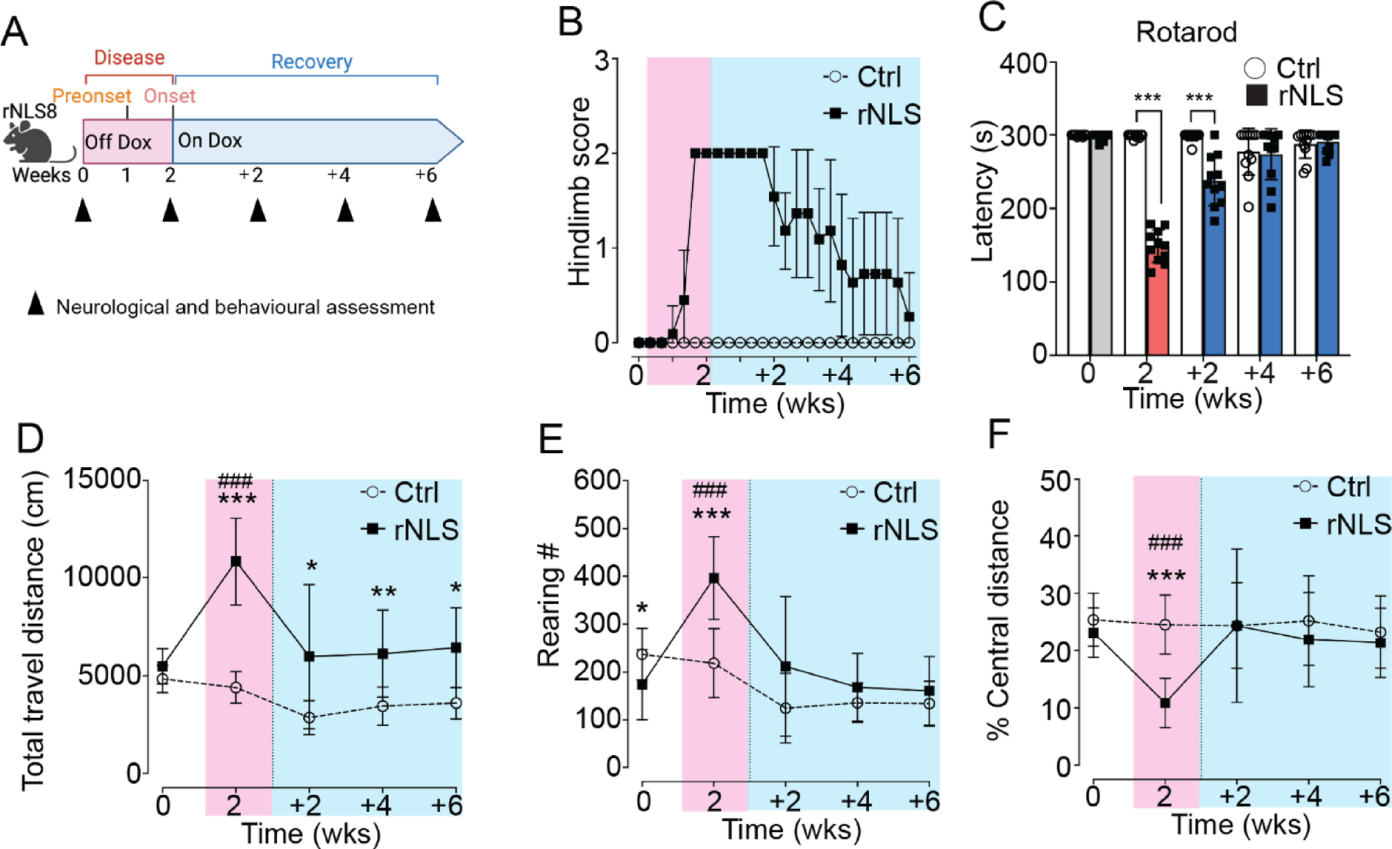
rNLS8 mice at disease onset show recoverable motor deficits but persistent hyperlocomotion. **A** Experimental schematic. Mice were behaviourally evaluated at 0 and 2 WOD, and +2, +4, +6 weeks back on dox. Results for the hindlimb neurological score **B** and latency to fall in seconds in the rotarod test **C** are shown. Mice that explored the open field arena for over 30 min were analysed for the total distance travelled **D**, rearing number **E** and relative central travel distance **F**. Data are shown as mean ± SD. Control (5 M, 6F), rNLS8 (3 M, 8F). *p < 0.05, *p < 0.01, ***p < 0.001 between control and rNLS8 mice at the same timepoints by repeated one-way ANOVA. ###*p* < 0.001 of rNLS8 mice relative to other timepoints by repeated one-way ANOVA

Synaptic dysfunction is increasingly recognised as a contributor to extra-motor phenotypes preceding neurodegeneration in ALS and FTD [13, 14]. Notably, TDP-43 pathology has been implicated in disturbances of synaptic function, neuronal activity, and behavioural alterations [14, 41]. To investigate whether behavioural changes are dependent on neuron loss or are triggered by the contribution of TDP-43 cytoplasmic mislocalisation to extra-motor symptoms prior to detectable neurodegeneration, we examined motor and extra-motor behaviours in “short-term diseased” rNLS8 mice induced to express hTDP-43^∆NLS^ for only 2 weeks off dox (WOD), a timepoint that demonstrates neuroinflammation [27] but is prior to loss of cortical or spinal cord neurons [28, 29], followed by 6 weeks back on dox to suppress transgene expression (Fig. 1A).

As expected, rNLS8 mice displayed a neurological score of 2 indicating collapsed hindlimb splay by 2 WOD, which recovered at 2 WOD + 6 weeks recovery (Fig. 1B). Likewise, motor performance on the rotarod test declined at 2 WOD and was fully restored by +4 weeks to a level indistinguishable from control mice (Fig. 1C). Overall, the motor behaviour results indicate early motor decline that is readily restored towards control levels over 6 weeks of recovery, similar to previous findings of motor function recovery when rNLS8 mice are returned to dox at later timepoints [28].

Having established the expected motor decline and recovery phenotypes in rNLS8 mice, we next initially assessed locomotor activity, anxiety-like behaviours, and general exploratory activities using the open field test [42, 43]. At 2 WOD, rNLS8 mice exhibited increased travel distance suggesting hyperlocomotion, and increased rearing frequency and decreased central travel distance, potentially indicating a disinhibition-like phenotype [42] (Fig. 1D–F). Further, rNLS8 mice remained significantly more hyperactive than controls across the recovery time course, with only partially normalised hyperlocomotion after 6 weeks back on dox (Fig. 1D), indicating enduring hyperlocomotion (Supplementary Fig. 1A). Interestingly, the increased rearing frequency (Fig. 1E) and decreased relative central travel distance (Fig. 1F) were completely restored to control levels by 2WOD + 6 weeks (Supplementary Fig. 1B, C). Overall, these findings suggest that the short-term hTDP-43^∆NLS^ expression was sufficient to induce motor and extra-motor behavioural phenotypes, some of which persisted in the recovery phase.

### Early and persistent differential exon usage in neuronal genes correlates with behavioural abnormalities in rNLS8 mice

**Fig. 2:**
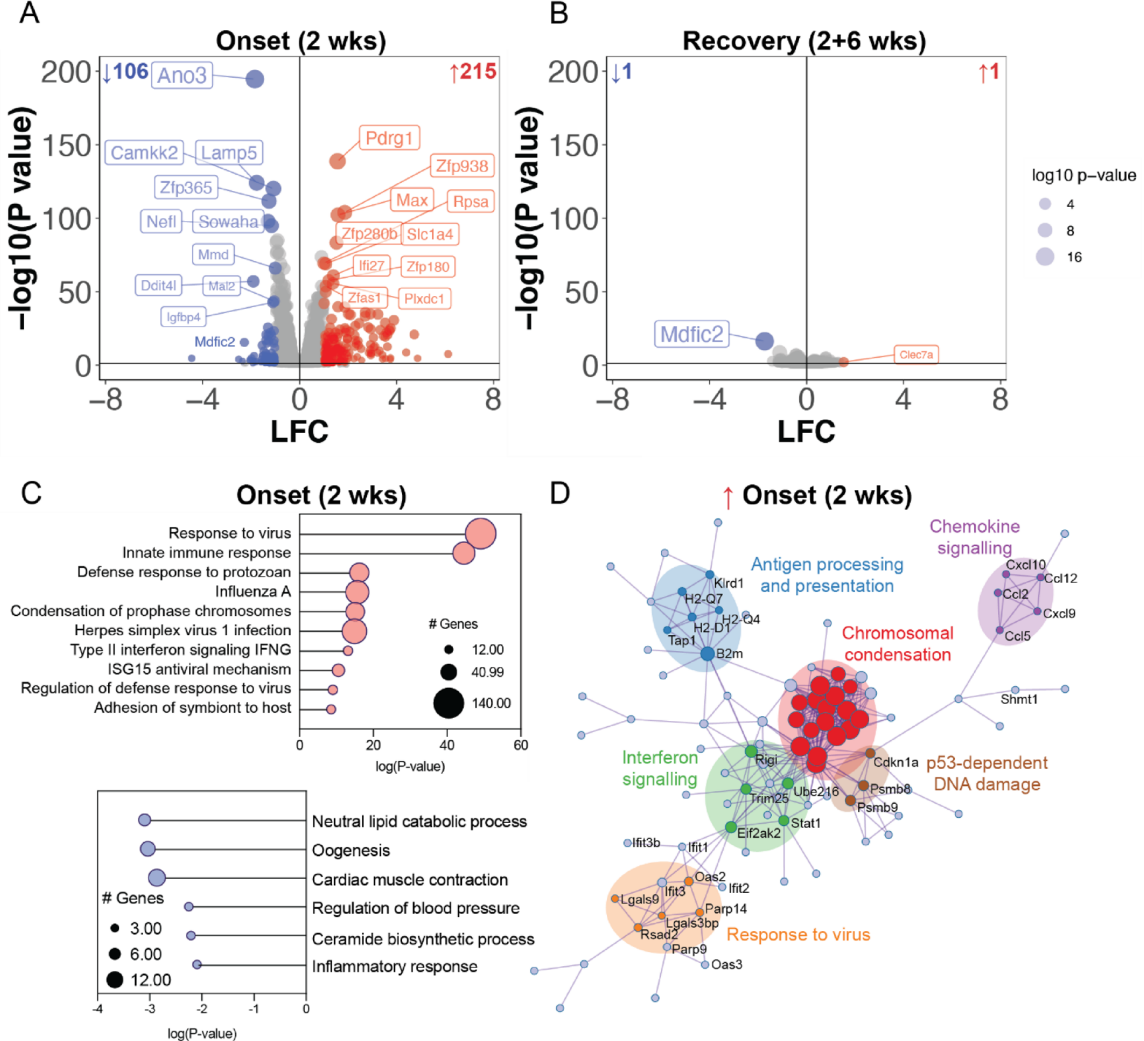
The cortical transcriptome at disease onset is strongly associated with neuroinflammation and this is completely normalised in recovery in rNLS8 mice. **A**, **B** Volcano plots of transcript mean log fold change (LFC; rNLS8/Con) and significance level [-log10(Pvalue)] of cortex from **A** disease onset (2 WOD) and **B** recovery (2 WOD + 6 weeks back on dox). Significantly upregulated genes are in red and downregulated in blue. **C** Gene ontology analysis of biological process enrichment at disease onset (2 WOD) of significantly upregulated (top) and downregulated (bottom) genes. Size of the data points are relative to the number of genes in the term. **D** Protein–protein interaction networks of significantly upregulated genes at disease onset (2 WOD). Genes are coloured by membership to given biological processes. Data are from *n* = 4 mice per group

To identify molecular changes associated with the enduring hyperlocomotion phenotype in the rNLS8 mice, we conducted bulk RNA sequencing of cortex tissue. Given that TDP-43 acts as an RNA-binding protein regulating both expression and splicing, we assessed changes to gene expression and differential exon usage (DEU; a measure of alternative splicing) in the cortex of mice at disease onset (2 WOD) and in the “short-term disease” recovery experimental paradigm (2 WOD + 6 weeks, Fig. 2).

At disease onset, *n* = 321 genes were significantly differentially expressed in the cortex (log fold change > 1, *P* < 0.05) including *n* = 106 downregulated and *n* = 215 upregulated (Fig. 2A, Supplementary Table 1). In contrast, after 6 weeks of recovery there were negligible transcriptomic differences between control and rNLS8 mouse cortex (Fig. 2B). Indeed, only *n* = 2 genes were persistently differentially expressed; *Clec7a* (encoding C-type lectin domain containing 7A) was upregulated and *Mdfic2* (encoding MyoD Family Inhibitor Domain Containing 2) was downregulated at both 2 WOD and 2 WOD + 6 weeks back on Dox. Differentially expressed genes at disease onset (2 WOD) were strongly associated with a decrease in lipid metabolism (lipid catabolic and ceramide biosynthetic processes, Fig. 2C) and an increase in inflammatory pathways (response to virus and innate immune response), with a highly connected protein–protein interaction network (Fig. 2D). The contrast in the number of differentially expressed genes between disease onset (2 WOD) and recovery (2WOD + 6 weeks) demonstrates the efficient regulation of the dox-suppressible hTDP-43^∆NLS^ transgene and the capacity of the cortex to recover to the control transcriptome. Nevertheless, there were no differentially expressed genes that persisted from onset to recovery from “short-term disease” that could explain the persistent hyperlocomotion phenotype in the rNLS8 mice.

**Fig. 3:**
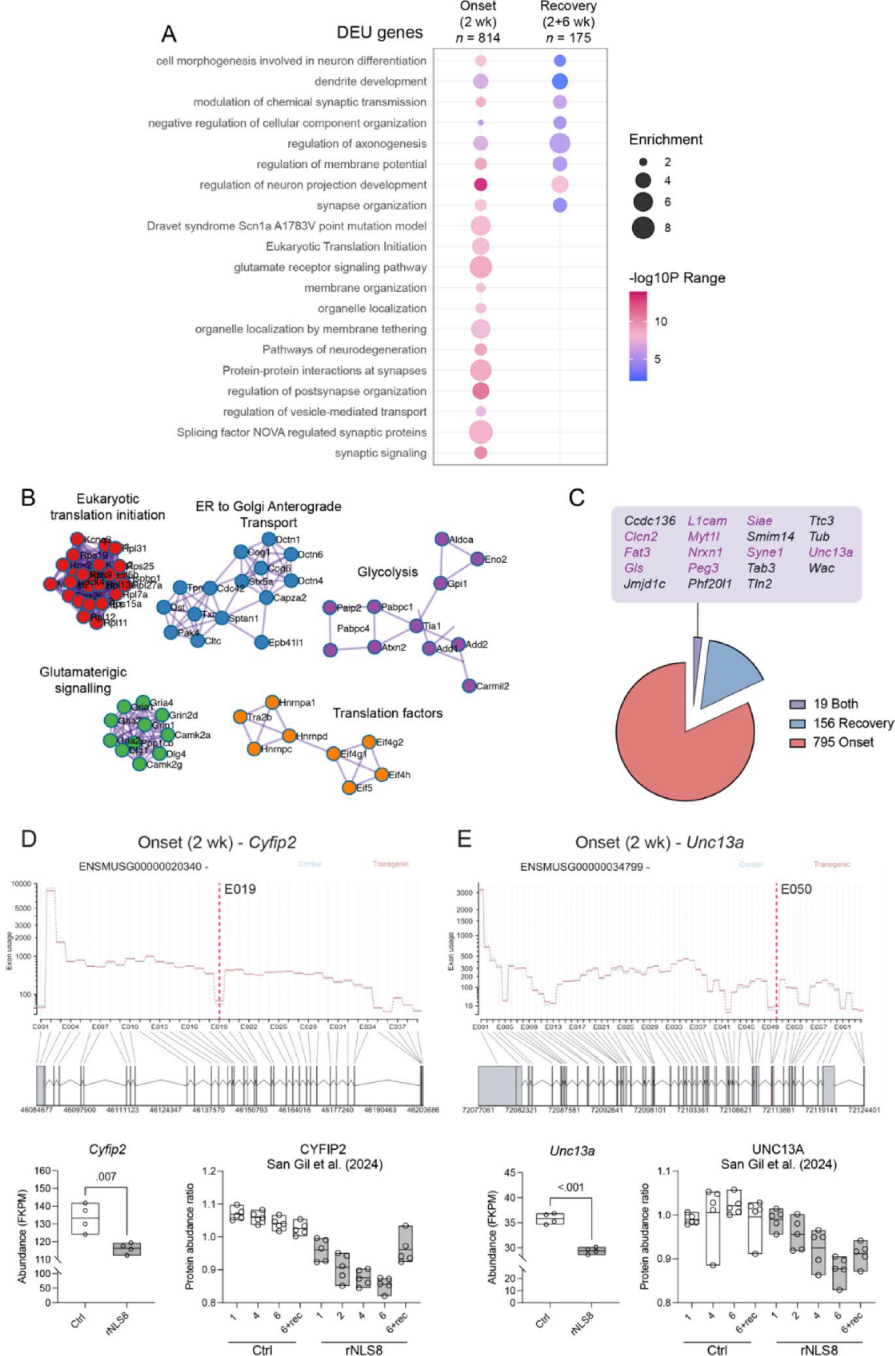
Differential exon usage in genes associated with axonogenesis and neuronal projection organisation at disease onset and in recovery cortex of rNLS8 mice. **A** Metascape gene ontology of onset (n = 814) and recovery (n = 175) genes that show significant alternative splicing. Bubble sizes increase with enrichment ratio and colour gradients are scaled to the range of P-values. **B** Protein–protein interaction networks of genes with significant DEU events. All significant networks relate to DEU genes in onset (2 WOD) cortex. **C** Number of genes with DEU events at onset (red), recovery (blue) or both onset and recovery (purple). List of genes with DEU events in both onset and recovery cortex are listed alphabetically and neuronal genes are in purple. **D** Differential exon usage of *Cyfip2* 019 correlates with a significant decrease in *Cyfip2* expression at 2WOD (fragments per kilobase of transcript per million; FKPM). Analysis of a published longitudinal proteomic dataset from control and rNLS8 cortex [29] shows a gradual decrease in cytoplasmic FMR1-interacting protein 2 (*CYFIP2*) abundance over time and partial recovery in rNLS8 mice allowed to recover for 2 weeks back on dox. **E**. Differential exon usage of Unc13a at E050 correlates with a significant decrease in Unc13a expression (FKPM) at 2WOD and a gradual decrease in UNC13A protein abundance over time [29]. Data represent *n* = 4–5 mice per group. *p < 0.05 by t-test

We then hypothesised that alternative splicing changes due to TDP-43 dysfunction may persist from disease onset to recovery to drive enduring behavioural changes in the rNLS8 mice. We identified *n* = 812 genes with significant differential exon usage (DEU; 1227 DEU events, *P* < 0.05) in the cortex of rNLS8 mice after only 2 weeks of hTDP-43^ΔNLS^ expression (Fig. 3). Genes with DEU events were enriched for cell projection organisation, synaptic signalling (including a significant network of glutamatergic synapse genes), and pathways involved in neurodegeneration such as ER to Golgi anterograde transport (Fig. 3A, B). Of these *n* = 812 genes, *n* = 522 (64.29%) were found to be known binding targets of mouse TDP-43 (Supplementary Table 1).

In the recovery cortex (2 WOD + 6 weeks), *n* = 175 genes showed significant DEU (199 DEU events, *P* < 0.05), which were enriched for neuron projection organisation, chemical synaptic transmission, and regulation of autophagy including TORC1 signalling (Fig. 3A). Interestingly, *n* = 156 genes were unique to the recovery cortex indicating DEU events in different genes after inhibition of neuronal hTDP-43^ΔNLS^ expression (Fig. 3C). Of the genes with DEU identified in the recovery cortex, *n* = 133 (76.9%) were found to be binding targets of mouse TDP-43, whilst 146 (84.4%) were found to be binding targets of human TDP-43 (Supplementary Table 1). We identified *n* = 19 genes with significant differential exon usage in both onset and recovery cortex, of which 16 are known binding targets of mouse TDP-43 (84.2%) and *n* = 10 are neuronal and/or synaptic genes, namely *Clcn2, Fat3, Gls, L1cam, Myt1l, Nrxn1, Peg3, Siae*, *Syne1* and *Unc13a* (Fig. 3C). Interestingly, the sites of DEU in these genes were different in onset and recovery cortex, with representative genes associated with ALS and/or FTD are illustrated in Supplementary Fig. 2.

Cell type enrichment analysis of genes with DEU events in recovery rNLS8 cortex (2 WOD + 6 weeks on dox) revealed pronounced CNS specificity (Supplementary Fig. 3). The most significant Allen Brain Atlas enrichments included vascular leptomeningeal cells (FDR = 1.79 × 10^−15^, OR = 7.27), microglia (FDR = 9.01 × 10⁻^15^, OR = 7.16), and oligodendrocytes (FDR = 4.46 × 10⁻^13^, OR = 5.81), with substantial gene overlaps (29–37 genes per term). Notably, established ALS risk genes including *Unc13a* (a major ALS susceptibility locus), *Kif5a* (involved in axonal transport dysfunction), *Cacna1a* (calcium homeostasis), and *Foxp1* (neuronal transcription factor) contributed to multiple significant neuronal and glial enrichments, demonstrating that genes with splicing alterations in recovery cortex are highly enriched for known ALS-relevant molecular pathways.

Most of the alternatively spliced genes did not show significant changes in protein abundance or transcript levels at disease onset (2 WOD; Supplementary Fig. 4A). Only two alternatively spliced genes (*Camkk2* and *Scn4b*) were downregulated and showed decreased protein abundance, while one alternatively spliced gene (*Shmt1*) was upregulated and exhibited increased protein abundance (Supplementary Fig. 4B) when compared to published proteomics data [29]. A subset of DEUs (4/19) in the persistent alternatively spliced genes were examined in different regions of the brain including cortex, hippocampus, and lumbar spinal cord (Supplementary Fig. 5). While all tested DEUs were validated in the rostral cortex of rNLS8 mice after 2 weeks of hTDP-43^ΔNLS^ expression, only *Myt1l* E035 was also significantly altered in both the hippocampus and lumbar spinal cord of these animals (Supplementary Fig. 5B). Expression of the *Syne1* isoform ENSMUST00000215887, which contained many significant DEUs, was significantly reduced in the hippocampus but unaltered in lumbar spinal cord of rNLS8 mice (Supplementary Fig. 5E).

Genes with DEU included *Unc13a* and *Cyfip2*, which have previously shown alternative splicing upon TDP-43 loss of function [19]*.* In contrast*,* the *Stmn2* gene that has previously shown alternative splicing in response to loss of TDP-43 [17, 20] was downregulated at onset (2 WOD) but normalised at recovery, without evidence of alternative splicing. DEU of *Cyfip2* (Fig. 3D) and *Unc13a* (Fig. 3E) correlated with a decrease in the expression of these genes in rNLS8 mouse cortex at 2WOD, and analysis of published longitudinal proteomics data from rNLS8 mouse cortex [29] showed a similar progressive decrease in the abundance of both proteins. These findings indicate that loss of TDP-43 function in the rNLS8 mice leads to alterations in gene expression and splicing reminiscent of human TDP-43 proteinopathies.

### rNLS8 mice develop hyperlocomotion, hyperactivity, and habituation deficits, concomitant with impaired motor functions, which persist even after motor recovery from advanced disease

**Fig. 4:**
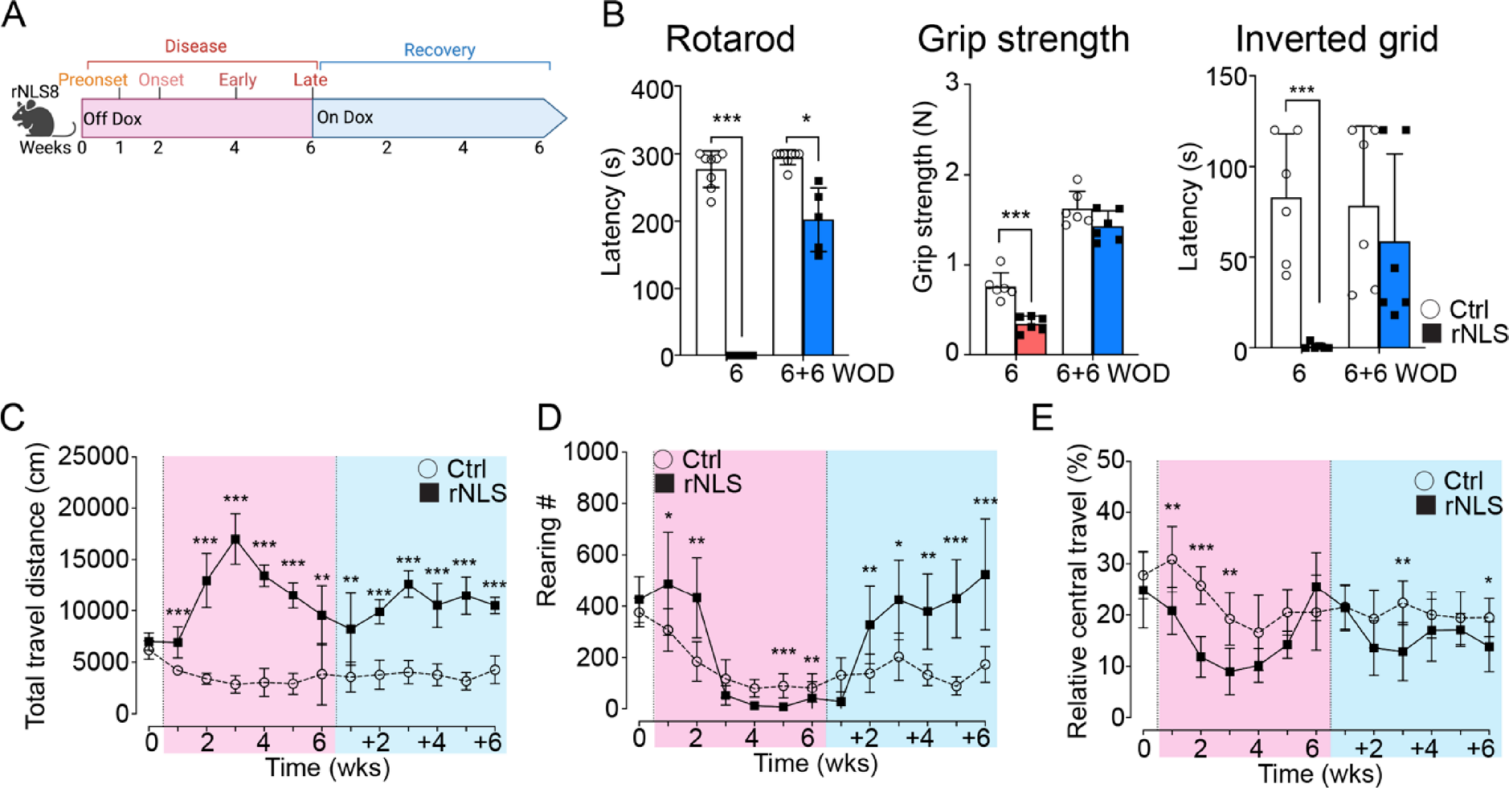
rNLS8 mice exhibit disinhibition-like phenotypes, and social and executive deficits despite impaired motor function. **A** The schematic of the rNLS8 mouse model. **B** The data of rotarod, grip strength and inverted grid tests at 6 WOD and 6 + 6 weeks back on dox (*n* ≥  6F per group). The open field analyses revealed the **C** total distance, **D** rearing number, and **E** relative central travel of the experimental mice that travelled in the open field arena over 30 min at baseline before the removal of dox (0 WOD), at 1-to-6 WOD and at additional 1-to-6 weeks back on dox. Control (8F), rNLS8 (6F). Data as mean ± SD. *p < 0.05, **p < 0.01 by two-way ANOVA

Given the indication of hyperlocomotion during recovery from early disease in rNLS8 mice but minimal persistent molecular changes, we next sought to evaluate how extra-motor phenotypes are affected in rNLS8 mice during recovery from late stage of disease, and to determine molecular correlates of these behavioural alterations. Before characterising the extra-motor behavioural phenotypes in the rNLS8 mice, we thus extended the disease phase up to 6 WOD, a timepoint that demonstrates significant neurodegeneration [24, 26, 28, 29] and neuroinflammation [26, 27, 29, 44, 45], prior to a recovery phase of an additional 6 weeks back on dox (Fig. 4A). As expected, rNLS8 mice showed significant motor deficits at 6 WOD, and importantly, recovered motor function in inverted grid performance and grip strength and dramatically improved rotarod performance through +6 weeks back on dox (Fig. 4B), aligning with previous reports [28].

In the open field test, the rNLS8 mice exhibited a significant increase in travel distance compared to the control mice at all timepoints examined, even prior to motor onset (1 WOD; Fig. 4C) and throughout recovery (+1 to +6 weeks back on dox). The hyperlocomotion in the rNLS8 mice peaked at 3 WOD (Fig. 4C), in line with a recent finding [24], then gradually decreased at later timepoints of disease correlating with increasing motor deficits in rNLS8 mice. In contrast to recovered motor function (Fig. 4B), the rNLS8 mice continued to display significant hyperlocomotion at all recovery timepoints (Fig. 4C). These results suggest the rNLS8 mice developed a disinhibition-like phenotype prior to the onset of motor deficits, which peaked at early disease stages, remained significantly increased despite severe motor deficits from 4 WOD, and then persisted during motor recovery. The rNLS8 mice also displayed dramatic increases of the travel distance in the outside areas (walls and corners) that involve exploring and walking [42] (Supplementary Fig. 6A, B), and persistent increases of initial total travel distance (0–5 min, Supplementary Fig. 6C), suggesting hyperactivity [42]. Furthermore, time-binned analysis revealed habituation deficits [43], at 4 WOD (5–30 min, Supplementary Fig. 6D).

rNLS8 mice also displayed a dramatic increase of rearing number in the open field test at 1 and 2 WOD (Fig. 4D) which was less evident at later timepoints when hindlimb function was impaired. Importantly, the rNLS8 mice re-displayed the increased rearing behaviour by 2 weeks back on dox (Fig. 4D and Supplementary Fig. 6E), indicating persistence of this phenotype that had been masked by motor impairment in the later disease stages. We also observed significant decreases in the relative central travel (%) in the rNLS8 mice after motor onset at 3 WOD, which persisted to the recovery phase through 6 WOD + 6 weeks, implying an anxiety-like phenotype (Fig. 4E). However, we did not observe significant space-related anxiety-like behaviours in the commonly used elevated plus maze paradigm (Supplementary Fig. 7). Nevertheless, we detected slightly decreased anxiety-like behaviour in rNLS8 mice in the light–dark transition test, with significantly decreased duration (%) in the dark chamber (Supplementary Fig. 8), in line with our previous finding in the *CAMKII*-hTDP-43^ΔNLS^ mice [33]. These data suggest the decreases of relative central travel (Fig. 4E) may be due to the dramatic increases of travel distance in the areas close to walls and corners. Together, the data revealed persistent hyperlocomotion and hyperactivity phenotypes in rNLS8 mice.

### rNLS8 mice display persistent social and executive deficits

**Fig. 5:**
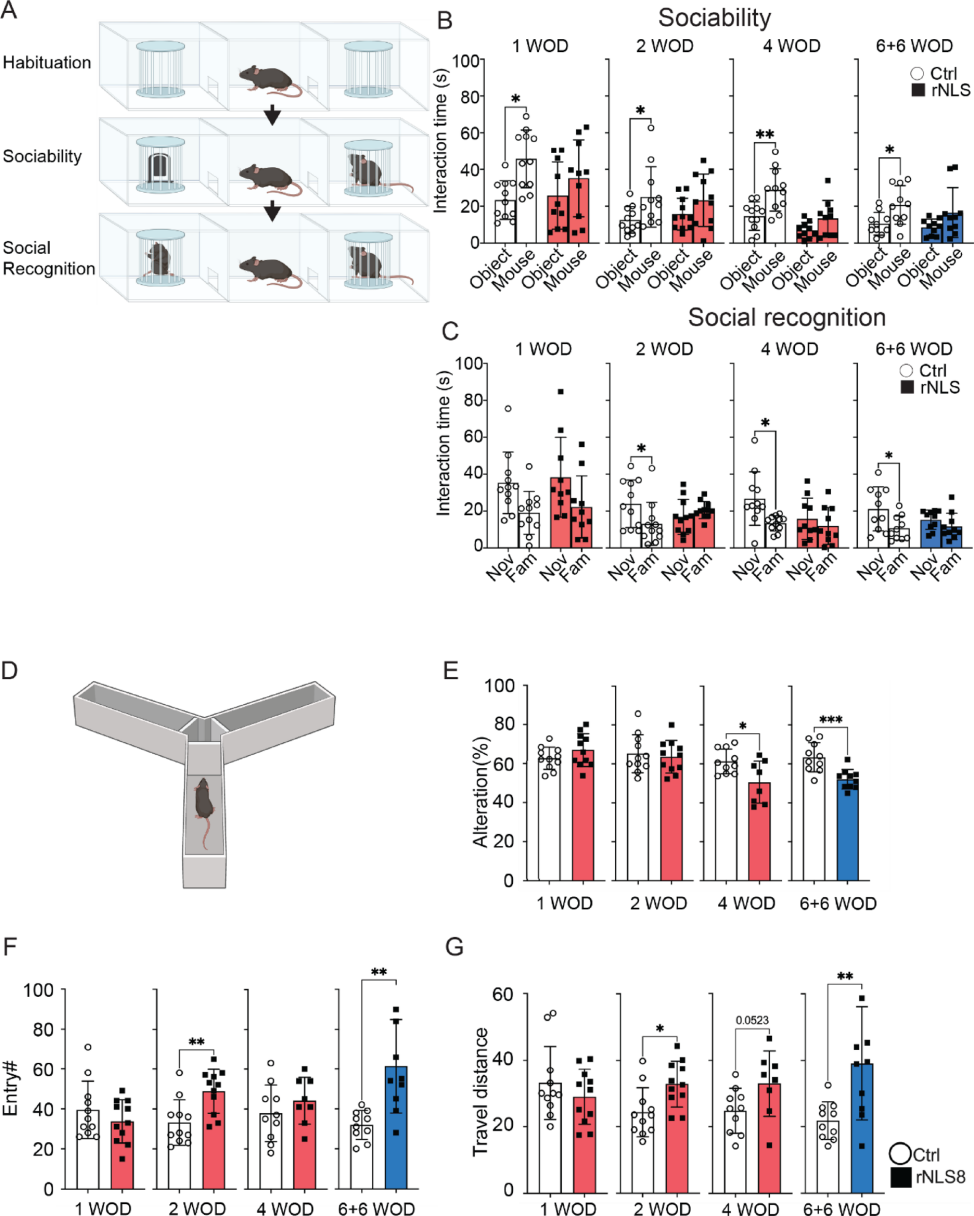
rNLS8 mice exhibit social and executive deficits despite impaired motor function. **A** Schematic of three-chamber social interaction tests**.** **B** Sociability session to compare the interaction time (in seconds) that the experimental mice spent with the non-social object (Object) versus the social object (Mouse) at 1, 2, 4 WOD and 6 WOD + 6 weeks back on dox**.** **C** Social recognition session to assess the interaction time (in seconds) that the experimental mice spent with a novel (Nov) mouse versus a familiar (Fam) mouse at 1, 2, 4 WOD and + 6 weeks back on dox**.** **D** Schematic of Y maze tests**.** **E** Spontaneous alteration (%), **F** total entry number, and **G** total travel distance (m) at 1, 2, 4 WOD and + 6 weeks back on dox. Control (5 M, 6F), rNLS8 (5 M, 6F). Data as mean ± SD. *p < 0.05, **p < 0.01 by two-way ANOVA

As apathy is a prevalent feature of individuals with ALS/FTD [8, 45], we examined sociability and social memory of rNLS8 mice using three-chamber social interaction tests [46] at 1, 2, 4 WOD and 6 WOD + 6 weeks back on dox (Fig. 5A). We did not include at 6WOD considering that severe motor impairments of rNLS8 mice, particularly in the hindlimbs, may compromise the assessment of cognitive and social behavioural outcomes in the social interaction test. As expected, control mice spent more time with an unfamiliar mouse compared to a non-social object, whereas rNLS8 mice did not show a preference for the unfamiliar mouse, suggesting impaired sociability in rNLS8 mice that persists in recovery (Fig. 5B). Control mice spent more time with a novel mouse than a familiar mouse (Fig. 5C), which indicates a natural preference [46]. In contrast, rNLS8 mice displayed similar length of interaction time between novel and familiar mice, suggesting impaired social memory. Importantly, the social memory impairment persisted at 6 WOD + 6 weeks back on dox (Fig. 5C), as did persistent increased travel distance in the testing arena correlating with our previous findings of hyperlocomotion (Supplementary Fig. 6). Overall, the social interaction results demonstrate persistent sociability and social memory deficits in rNLS8 mice in disease and despite motor function recovery.

We further examined spatial working memory to assess executive function in rNLS8 mice, using the Y-maze test (Fig. 5D) as in our previous reports of *CAMKII*-hTDP-43^ΔNLS^ mice [30, 33]. Similar to social interaction test, we did not include 6WOD time point in the Y-maze test due potential impact of the severe motor deficit in the rNLS8 mice. rNLS8 mice displayed similar spontaneous alterations (> 60%) to the control mice until disease onset, including 1 and 2 WOD (Fig. 5E). Interestingly, the rNLS8 mice started to exhibit a significant reduction of spontaneous alteration (%) comparing to controls at 4 WOD, in line with a recent report suggesting contribution of hippocampal neurodegeneration to executive deficits in the rNLS8 mice [24]. Notably, the executive deficits persisted to the recovery stage in the rNLS8 mice (Fig. 5E). Moreover, we observed the consistent increased entry number (Fig. 5F) and total travel distance (Fig. 5G) at 2 WOD and 6 WOD + 6 weeks back on dox, respectively, in line with the findings in the open field and social interaction tests. It is worth further investigation as to how neurodegeneration in different brain regions contributes to executive dysfunction in the rNLS8 mice and whether this is related to ALS and FTD.

### Glutamatergic signalling protein loss persists in the rNLS8 mouse cortex following motor recovery, similar to changes in human ALS/FTD

**Fig. 6:**
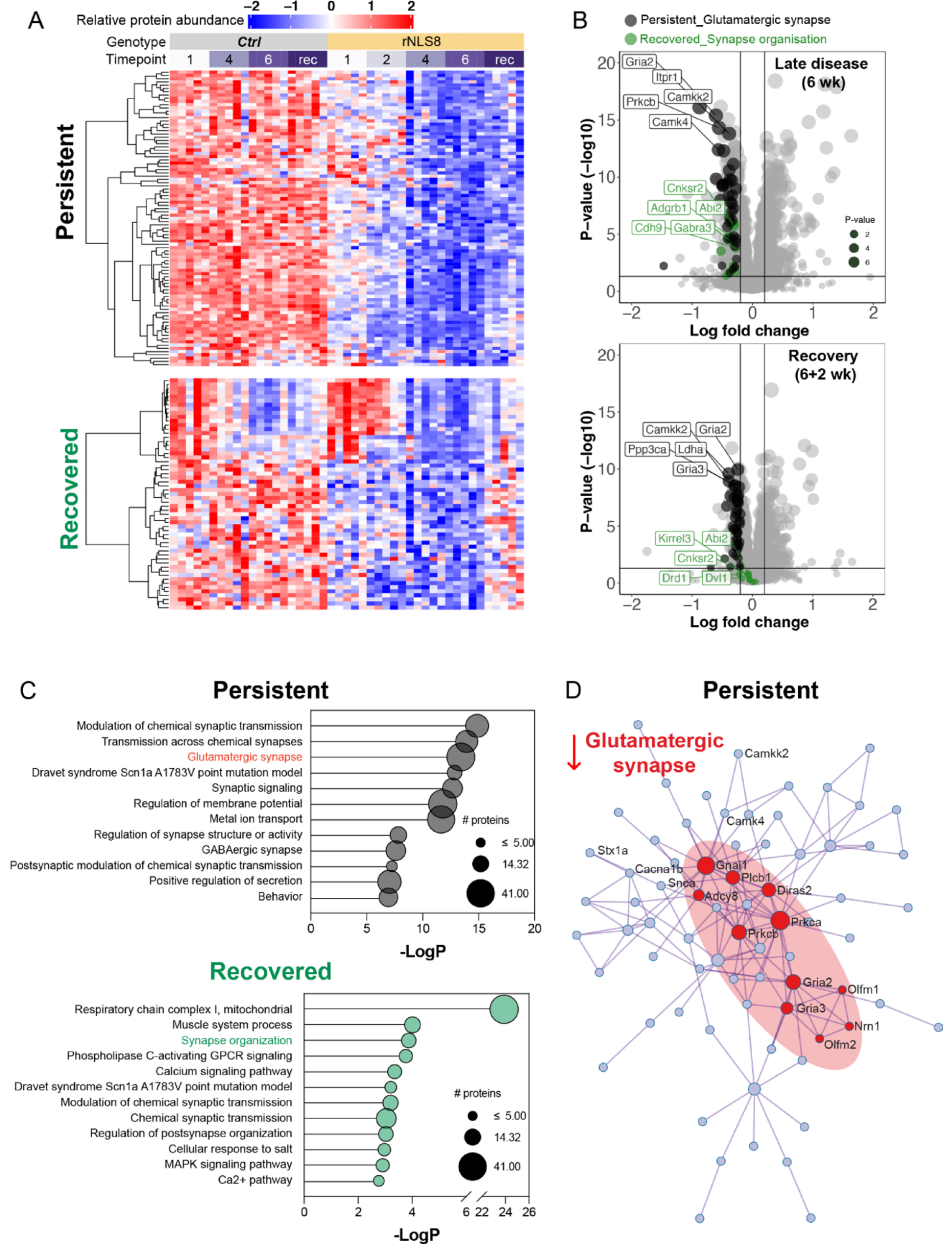
Depletion of glutamate signalling proteins persists in rNLS8 mice despite motor recovery. **A** Heatmap of the relative protein abundance of quantified proteins in “persistent” and “recovered” protein subsets in rNLS8 mice at 1, 2, 4, and 6 WOD (WOD) and in recovery (6 WOD + 2 weeks on dox) compared to littermate controls (Ctrl). The persistent subset of proteins represents those that are significantly decreased (fold change > 1.2, *p* < 0.05) in rNLS8 mice in late disease (6 WOD) and recovery (6 WOD + 2 weeks on dox). The recovered subset of proteins represents those that are significantly decreased in rNLS8 mice in late disease (6 WOD) but are not significantly different to control levels in recovery (6 WOD + 2 weeks on dox). Each column represents data from an individual mouse (*n* = 5/group), and red = high and blue = low relative protein abundance. **B** Volcano plots of mean log fold change (rNLS8/Con) and *p* (− log10) from late disease (6 WOD) and recovery (6 WOD + 2 weeks on dox) timepoints. All proteins (grey), proteins belonging to the glutamatergic synapse (black) gene ontology and the synapse organisation (green) gene ontology terms are shown. (**C**) Metascape gene ontology analysis of persistent and recovered subsets of proteins. The size of the bubble indicates the number of proteins in each term. (**D**) Protein–protein interaction network of components of the glutamatergic synapse (red), which are persistently significantly decreased in disease (6 WOD) and recovery (6 WOD + 2 weeks on dox). Proteomics data [29] were re-analysed to identify persistently decreased and recovered proteins in the rNLS8 mouse cortex

We hypothesised that synaptic mechanisms [14, 47, 48] may contribute to the persistent behavioural changes in the rNLS8 mice, and we therefore re-analysed a previously published longitudinal proteomics dataset of the rNLS8 mouse cortex [29]. We focused on the subset of proteins that were significantly decreased in the cortex in late disease (6 WOD, *n* = 305 proteins; fold change > 1.2 and *P* < 0.05), which were enriched for biological processes associated with “chemical synaptic transmission” and “behaviour”. Approximately half of this subset of late disease decreased proteins (*n* = 117; 51%) were restored to control levels in recovery (“recovered” proteins = down at 6 WOD but no change at + 2 weeks back on dox), and the other half remained decreased in recovery in rNLS8 mice (*n* = 114, 49%; “persistent” proteins = down at 6 WOD and down at + 2 weeks back on dox; Fig. 6A and B). Interestingly, “recovered” and “persistent” subsets of proteins were both enriched for proteins associated with synapse biology (Fig. 6C, Supplementary Table 2), suggesting that some synaptic proteins are more “plastic” and capable of restoring to control levels than others, for example, recovered synaptic gamma-aminobutyric acid receptor subunit alpha-3 (GABRA3) that was altered in sporadic FTD-TDP cases [49]. Interestingly, persistently decreased proteins in disease and recovery were enriched for glutamatergic synapse proteins, revealed by two independent gene ontology analysis approaches and a large protein–protein interaction network (Fig. 6C and D, Metascape and Ingenuity Pathway Analysis; Supplementary Table 2). Persistently decreased glutamatergic synapse proteins included syntaxin-1A (STX1A), voltage-dependent N-type calcium channel subunit alpha-1B (CACNA1B), and calcium/calmodulin-dependent protein kinase type IV (CAMK4), as well as glutamate ionotropic receptor AMPA type subunit 2 and 3 (GRIA2 and GRIA3). SynGo analysis suggested alterations in both pre- and post-synaptic components (Supplementary Fig. 9).

**Fig. 7:**
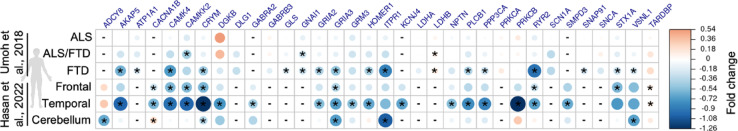
Glutamatergic synapse proteins that are persistently decreased in rNLS8 mice are also significantly decreased in human ALS and FTD post-mortem tissues. A subset of *n* = 40 persistent significantly decreased proteins from the rNLS8 cortex “glutamatergic synapse” gene ontology term were input into the TDP-map webtool [29]. A total of *n* = 33 proteins/genes were detected in the transcriptomic [40] and proteomic [39] datasets of human post-mortem brain tissue and abundance is plotted. Datapoints are colour-scaled to illustrate the log fold change where red is increased and blue is decreased compared to controls. Circle size is dependent on the magnitude of log fold change, the larger the change the larger the circle size. A (*) indicates significance (*P* < 0.05) and (-) indicates that the protein/gene was not detected in the datasets

A proportion (67.5%) of the glutamatergic synapse proteins (*n* = 40 proteins) that were persistently significantly decreased in rNLS8 mouse cortex in late disease and recovery were also significantly decreased in human ALS and FTD post-mortem brain tissues (*n* = 33 detected, *n* = 27 significantly decreased) [39, 40]. This was revealed by targeted re-analysis of published proteomic and transcriptomic datasets using the webtool “TDP-map” (Fig. 7 and Supplementary Fig. 10) [27–29]. Together, these data show that a subset of disease-relevant glutamatergic synaptic proteins that are depleted in rNLS8 mouse cortex correlating with persistent extra-motor phenotypes, are also affected in human TDP-43 proteinopathies.

## Discussion

The growing recognition of extra-motor symptoms associated with ALS highlights their significant impact on quality of life and disease prognosis. This raises the need for understanding of the mechanisms behind these symptoms, as well as the development of animal models that accurately reflect the complexities of ALS. Here, we demonstrated that expression of disease-reminiscent hTDP-43^∆NLS^ in neurons leads not only to ALS-like motor decline but also to persistent extra-motor behavioural impairments in rNLS8 mice. These persistent behavioural deficits associate with changes in alternative splicing of neuronal genes and levels of glutamatergic synapse proteins, suggesting potential mechanisms of relevance to disease.

### Early and persistent extra-motor behaviours in rNLS8 mice and human disease

rNLS8 mice have been well characterised as a model developing motor deficits [26–28, 50, 51], and recent studies have suggested that rNLS8 mice also develop extra-motor phenotypes including hyperactivity and anxiety deficits in early disease stages [24]. Our study reveals that rNLS8 mice display many early extra-motor behaviours that are readily detectable despite motor impairments, and further that these changes persist despite recovery of motor function upon suppression of the hTDP-43^∆NLS^ transgene. Notably, phenotypes such as hyperlocomotion and social withdrawal emerge early, prior to the onset (1 WOD) of detectable motor impairments. Importantly, our findings reveal that extra-motor behaviours persist even during the recovery phase, when motor function, muscle innervation, and overall survival otherwise improve [28]. This observation suggests that TDP-43 pathology rapidly inflicts lasting damage on neural circuits, possibly through ongoing synaptic dysfunction at both transcriptomic and proteomic levels [29] (Fig. 3 and 6). While neuronal loss in vulnerable populations, such as layer V cortical neurons [28] and CA3 hippocampal neurons [24], may contribute to these persistent deficits, the enduring synaptic impairments that we detected via transcriptomic analysis indicates these deficits may be caused by circuit changes rather than simply due to frank neurodegeneration.

Our results provide valuable insights that align with the extra-motor phenotypes observed in various other TDP-43 mouse models. For example, brain-specific expression of hTDP-43^∆NLS^ driven by the *CAMKII* promoter induces hyperlocomotion and social withdrawal in mice [30, 33]. Similarly, overexpression of wildtype TDP-43 in the forebrain leads to progressive motor and memory deficits [33]. Additionally, TDP-43 mutant knock-in mice exhibit various behavioural alterations, highlighting the contributions of both gain- and loss-of-function mechanisms contributing to motor and extra-motor dysfunctions. For instance, TDP-43^G348C^ mutant mice show discernible motor and memory deficits [50], whereas TDP-43^Q331K^ mutants present cognitive and motor impairments [51, 52]. Conversely, TDP-43^M337V^ and TDP-43^G298S^ mutants do not exhibit significant cognitive or behavioural changes [53], highlighting the need to carefully draw conclusions from studies of different models. Notably, the extra-motor phenotypes in rNLS8 mice align with other ALS/FTD mouse models that exhibit TDP-43 pathology, such as C9ORF72 repeat expansions (G4C2)66 mice [54], C9BAC mice [54], Fus^ΔNLS/+^ mice [55], and rAAV2/8-UBQLN2 mice [56]. These findings suggest a potentially universal mechanism involving TDP-43 pathology that contributes to extra-motor dysfunction in ALS/FTD. Notably, unlike other TDP-43 models where extra-motor symptoms typically develop only after prolonged disease progression, for example TDP-43^M337V^ and TDP-43^G298S^ mice that show motor neuron degeneration only after 2.5 years [53], the rNLS8 mouse model exhibited a rapid onset of both motor and extra-motor phenotypes.

### Synaptic mechanisms in TDP-43-associated disease

Early extra-motor changes in rNLS8 mice correlated with significant early decreases in levels of proteins associated with synaptic transmission and organisation. Cortical hyperexcitability has been long proposed as a causative mechanism for ALS and FTD associated not only with TDP-43 [3, 29, 57] but other disease proteins for instance C9ORF72 [48] or FUS [58]. Notably, hyperexcitability is also a key feature of ALS [59]. In this study, we identified a hyperlocomotion and hyperactivity phenotype in the rNLS8 mice, which may be linked to early increased neuronal activity in cortical regions [27], preceding neurodegeneration at 3-4WOD [24, 27, 28]. Interestingly, both excitatory and inhibitory neurons displayed increased neuronal activity in layer V and layer II/III of rNLS8 mice [27], supporting the idea that the imbalance of excitatory and inhibitory regulation contribute to disease development in the ALS [59]. A recent report [24] showed significant hippocampal hyperexcitability in the of rNLS8 mice, implying TDP-43 pathology disrupts multiple brain regions and global circuitry. Moreover, similar hyperactivity was noted in the cortical brain section in *CAMKII*-hTDP-43^ΔNLS^ mice [60], emphasising the importance of exploring how TDP-43 pathology may lead to both gain- and loss-of-function that could result in hyperexcitability and consequent neurotoxicity [41].

TDP-43, as a DNA-/RNA-binding protein, binds the transcripts of numerous synaptic genes that regulates neural circuitry vital for the cognitive and motor functions under health and disease conditions [53–55]. The role of TDP-43 in RNA metabolism contributes to disease, and targeting TDP-43 pathology can ameliorate the disease development and prolonged the life span [25]. TDP-43 loss has been implicated in the regulation of key synaptic proteins, including syntaxin-binding protein 1 (*STXBP1*), synaptotagmin-7 (*SYT7*), and *UNC13A* [19, 61]. Notably, given that cryptic splicing of *Unc13a* results from loss of functional TDP-43 [19], correcting *Unc13a* splicing presents a promising therapeutic approach.

Our data reveal that the rNLS8 cortex displays alternative splicing changes, a hallmark of loss of TDP-43 function. Alternatively spliced genes were enriched for axonogenesis and chemical synaptic transmission, with a subset of genes retaining DEU events during recovery phase. Interestingly, many of the genes retaining DEU events are known binding targets of TDP-43, with the majority of the genes identified in the onset and recovery cortex, respectively, found to be splicing targets of mouse TDP-43. It is possible that the alterations observed could arise directly from TDP-43 dysfunction, either mislocalised human TDP-43 expression or downregulated expression of nuclear mouse TDP-43 in the rNLS8 cortex. Several DEU events in the persistent alternatively spliced genes were enriched in the cortex but were not significantly altered in the hippocampus and lumbar spinal cord, suggesting that splicing events in these genes may contribute to the persistent behavioural phenotypes observed in these mice. A recent study also showed that rNLS8 mice develop alterations in 3’ untranslated region polyadenylation, affecting genes crucial for synaptic function and showing resemblance to human ALS/FTLD-TDP [23]. Together these data establish that loss of normal TDP-43 function contributes to the molecular signature of the rNLS8 mouse cortex.

Our study further highlights a progressive decrease in proteins related to chemical synaptic transmission in the rNLS8 cortex prior to disease onset, with particular susceptibility observed in glutamatergic synapse proteins [29]. These glutamatergic synapse proteins were particularly susceptible and significantly decreased from disease onset (1 WOD) and remained decreased in the recovery phase. This finding aligns with our previous biochemical results that cytoplasmic TDP-43 mislocalisation leads to decreased protein levels of AMPAR subunit in the rNLS8 [62] and *CAMKII*-TDP-43^ΔNLS^ mice [60]. While we have explored targeting AMPAR using riluzole, which does not effectively prevent disease phenotypes in rNLS8 mice [62], alternative pharmacological or genetic strategies to modulate glutamatergic signalling merit investigation for therapeutic development. Targeting glutamatergic receptors could enhance synaptic activity and thereby improve cognitive function in neurodegenerative disease [63]. In addition, rNLS8 mice exhibit astrogliosis [28, 29] and activated astrocytes, a consistent inflammatory feature in ALS [64] thathave been linked to both the pathology and motor deficits in ALS patients and TDP-43 mouse models [64–66]. The role of astrocytes in glutamate reuptake at synapses is critical for maintaining normal brain function and preventing excitotoxicity associated with disorders like ALS/FTD [64, 66]. Notably, the relationship between astrocytic modulation of synaptic function and behavioural changes, like neuronal hyperactivity and hyperlocomotion [65], further underscores the potential impact of astrocytic dysfunction in addition to neuronal synaptic alterations on disease-relevant phenotypes in rNLS8 mice and ALS patients. These findings may suggest that ALS pathology induces a coordinated loss of TDP-43-dependent gene repression across multiple CNS cell types, with the resulting splicing dysregulation persisting even after apparent recovery. Future studies could examine the underlying mechanisms using optogenetic or electrophysiological techniques.

### Limitations

The impact of TDP-43 pathology on different brain regions in addition to the cortex warrants further exploration. For example, recent studies suggest that TDP-43 dysfunction leads to hippocampal neuron loss in rNLS8 mice [24] and substantial cerebellar involvement in human postmortem tissue [67], indicating that neurodegeneration of distinct neuronal subpopulations may contribute to the various behavioural phenotypes of this model. Further, consideration of differences between mouse and human pathophysiology is important. Recognising the inherent limitations of translating findings from animal models to human conditions, we note that von Economo neurons and fork cells that are key targets of degeneration in human FTD that may play a role in development of disease-relevant behavioural dysfunctions [68], are considered absent in mice. Additionally, the genes containing UG-rich TDP-43 RNA binding sequences, and that are therefore splicing targets of TDP-43, show differences between mouse and human [69]. However, in this study we detected changes in levels of splicing and protein of key TDP-43-related loss of function targets, including *Unc13a*. Interestingly, UNC13A protein levels were decreased in the rNLS8 mice similar to decreases seen in human disease, despite different *Unc13a* splice sites in human and mouse transcripts due to species-specific TDP-43 binding motifs. In addition, our studies of persistent behaviours and synaptic gene/protein changes extended only to six weeks after inhibition of the hTDP-43^∆NLS^ transgene, and studies at further timepoints are warranted to examine the dynamics of neural recovery processes. Lastly, we acknowledge that the current sample sizes were influenced by breeding logistics and animal availability, resulted in unbalanced numbers of each sex per group, which may limit the statistical power to detect sex-related effects. Future studies therefore could deliberately examine sex as a biological variable contributing to potential sex differences in the behavioural changes of this model during disease and recovery phases.

## Conclusion

Our study expands the known molecular and behavioural alterations that result from accumulation of cytoplasmic TDP-43 in neurons through a comprehensive integration of behavioural, transcriptomic, and proteomic analyses. Our findings reveal that synaptic dysfunction, particularly at glutamatergic synapses in the rNLS8 mouse cortex, is associated with enduring behavioural deficits. Importantly, our results indicate that while recovery of motor function can be achieved rapidly with TDP-43 pathology clearance, extra-motor behavioural phenotypes and mis-splicing of RNA persist. This work expands the behavioural approaches that may be applied in rNLS8 mice for pre-clinical assessment of potential ALS and FTD therapeutics. Our work also underscores the need for further exploration of the mechanisms driving TDP-43-mediated neurodegeneration and highlights the importance of understanding the mechanisms of synaptic dysfunction in disease. These findings may also inform the development of therapeutic strategies to address the management of extra-motor symptoms to enhance the quality of life of individuals affected by these diseases.

## Supplementary Information

Supplementary Material 1

Supplementary Material 2

Supplementary Material 3

## Data Availability

The transcriptomic data has been deposited to the Sequence Research Archive (PRJNA1298393). Previously published mouse proteomics 29, human proteomics 39 and human transcriptomics 40 datasets used in this work for comparative analyses are available from the respective references. Supplementary Data are provided with this paper and any additional information required to re-analyse the data reported in this paper is available from the lead contact upon request.
