# Supplementary material for: Synaptic changes contribute to persistent extra-motor behaviour deficits in amyotrophic lateral sclerosis
Source: Acta Neuropathol Commun. 2025 Dec 21;14:26. doi: 10.1186/s40478-025-02150-5 (PMC12837556; doi:10.1186/s40478-025-02150-5)
Supplement: Supplementary file 1 — Supplementary Material 1 [file 40478_2025_2150_MOESM1_ESM.pdf]

Supplementary Material

Synaptic changes contribute to persistent extra-motor behaviour deficits in amyotrophic lateral sclerosis

Wei Luan[1*], Rebecca San Gil[1,2,3*#], Lidia Madrid[1], Maize C. Cao[4], Florencia Vassallu[5,6], Juliana Venturato[1,3,9], Phillip K. West[2,3], Heledd Brown-Wright[1], Adekunle T. Bademosi[1], Yi Jia Chye[1,2,3], Hao Yu Wu[1], Anna Harutyunyan[3,9], Katherine J. Robinson[3,9], Mu Sheen Chang[1,3,9], Catherine A. Blizzard[7,8], Emma L. Scotter[4], Lionel M. Igaz[5,6], Adam K. Walker[1,3,9#]

* These authors contributed equally to this work

[#]Correspondence:

adam.walker@sydney.edu.au and rebecca.sangil@sydney.edu.au

Supplementary Methods and Materials

Elevated plus maze test

Anxiety-like behaviour was assessed as described (Alfieri JA 2016) using an elevated plus maze consisting of two open arms (30 cm × 6 cm × 0.3 cm) and two closed arms (30 cm × 6 cm × 15 cm) with opaque walls. The apparatus was elevated 40 cm above the floor, and the duration of the test was 5 min. The maze was placed in the centre of a homogenously illuminated room (2 m × 1.8 m; 100 lux across arms). At the beginning of the test, mice were placed in the central square facing the open arm opposite to the investigator. Number of open arm entries, percentage of time in open arms, total arm entries and total distance travelled was measured.

Light-dark transition test

The open field chamber as mentioned above was divided equally into the light side that was brightly illuminated by white diodes (390 lux), and the dark side that was is illuminated at 2 lux. Mice are placed into the arena facing the dark side and the door is opened automatically 3 seconds after the mouse is detected by the infrared camera. The door is used so that the mice do not enter the light chamber immediately after the release with their motivation to escape from experimenter, since the latency to enter the light chamber may serve as an index of anxiety-like behaviour. Animals were placed toward the dark chamber and allowed to freely explore for 30 min. The distance travelled in each chamber, the total number of transitions, the time spent in each chamber, and the latency to enter the light chamber recorded by EthoVision XT software.

Cell-type enrichment analysis

Cell type enrichment analysis was performed using the EnrichR web platform[1, 2] to identify over-representation in cell types among the persistently alternatively spliced genes. Over-representation analysis was conducted against the Allen Brain Atlas 10x scRNA-seq 2021[3] dataset. Statistical significance was assessed using Fisher's exact test with Benjamini-Hochberg false discovery rate (FDR) correction applied across all tested gene sets within each database. Enrichment terms with FDR-corrected p-values < 0.05 were considered statistically significant. Combined scores were calculated as the product of -$\log_{10}$(p-value) and z-score to provide a unified metric for enrichment strength, and odds ratios were computed to quantify the magnitude of over-representation relative to background gene sets.

Real-time quantitative PCR (QPCR) assay

RNA was extracted from the right rostral cortex, hippocampus, or lumbar spinal cord with Qiazol (Qiagen #79306) using the Qiagen RNeasy Mini Kit (Qiagen, #74104) and Precellys tissue homogeniser (Bertin Instruments, Montigny-le-Bretonneux, France). On-column DNase I digestion was conducted using RNase-free DNase I (Qiagen #79254). The purity and concentration of extracted RNA was determined using a Nanodrop-2000 spectrophotometer (Thermo Fisher). cDNA was synthesised from 1 µg total RNA using SensiFAST™ cDNA Synthesis Kit (Meridian Bioscience #BIO-65054). The QPCR was set up using SensiFAST™ SYBR® No-ROX (Meridian Bioscience #BIO-98005), 400 nM primer pairs, and 40 ng template cDNA and was performed with a LightCycler® 480 (Roche, Basel, Switzerland) using 2-step cycling (95°C for 2 min, then 40 cycles of 95°C for 5 sec then 60°C for 30 sec, followed by

melt curve analysis). The relative changes in gene expression were calculated based on the housekeeping gene *Gapdh* according to the $2^{-\Delta\Delta Ct}$ method and normalised to control groups. Primer sequences: *Camkk2* (forward: CAACGTGGTGAAGCTGGTAGAG; reverse: TGGTCTTCGGACAGTGGCTTGA), *Scn4b* (forward: GGCAGATACACCTGCTTCGTGA; reverse: TGAGAGTCACCGTGTTGTCCAC), *Shmt1* (forward: CTGGAGATGCTGTGTCAGAAGC; reverse: TGAGGCTCTACCAGGGCAGTAT), *Tab3* E008 (forward: AGTGGTGCCAGCATTTTCTT; reverse: ACTCTGTCATTTTCGTCAACGTC), *Myt1l* E035 (forward: CCTGGCGGACAAAAGCATTC; reverse: ACTGAACATGAGCAGGTGGT), *L1cam* E024 (forward: ATCACATGGAAGCCCCTTCG; reverse: GGCCAGGCTCACTCCAATAC), *Syne1* E127 (forward: CCTGTCATCAGAGTTGGAGCA; reverse: GACACAGGAGACACTATTGGGA), *Syne1* ENSMUST00000215887 (forward: ATGCTTCTCAGCCTCTTGGTAA; reverse: TAAGTGCAGCTCGGATGTGG), and *Gapdh* (forward: GCACAGTCAAGGCCGAGAAT; reverse: GCCTTCTCCATGGTGGTGAA).

Supplementary Figures

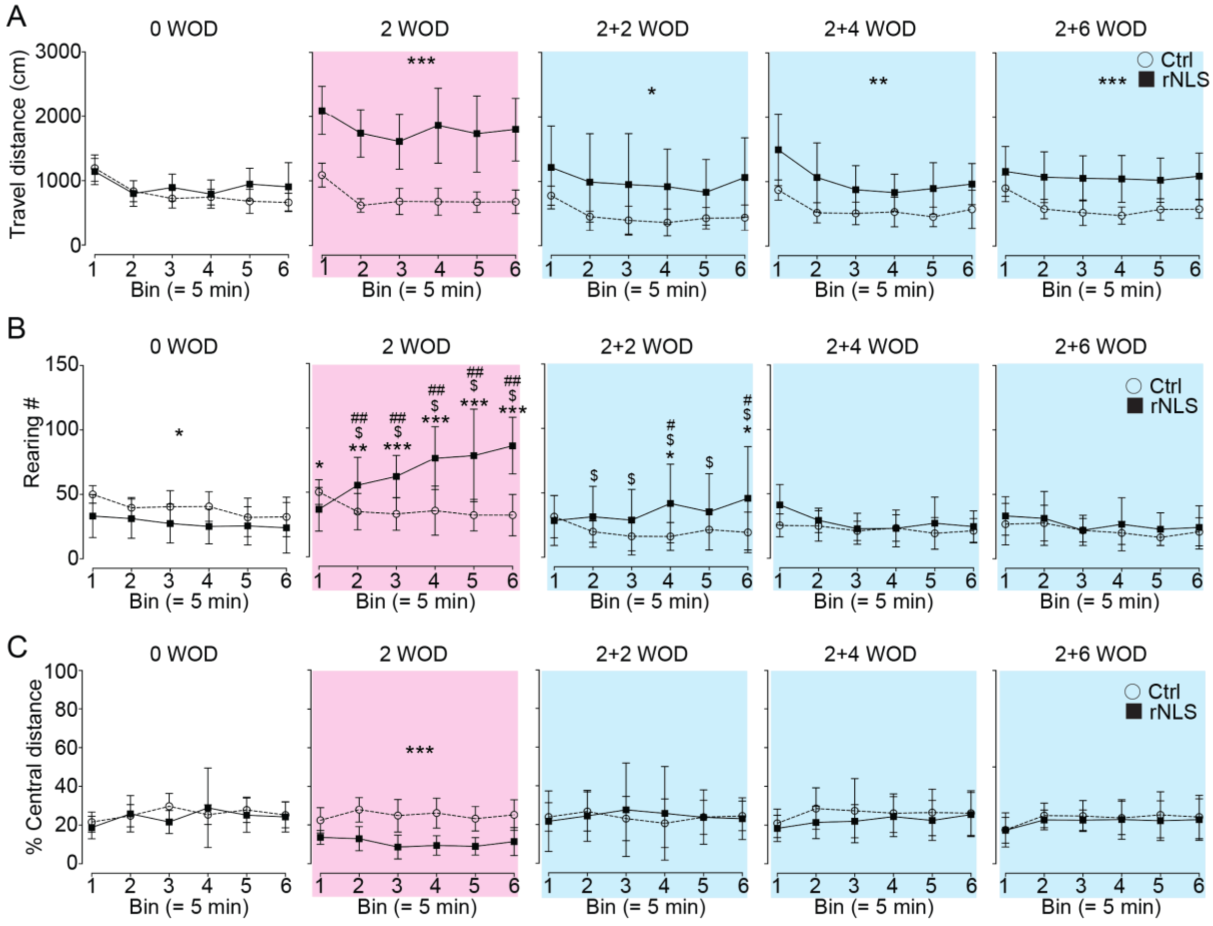

Supplementary Figure 1. Open field analysis of rNLS8 mice over weeks. A*. The travel distance (cm) per bin (= 5 min) that the experimental mice travelled in the open field arena at 0 WOD, 2 WOD, 2 WOD + 2 weeks back on dox , 2 WOD + 4 weeks back on dox, and 2 WOD + 6 weeks back on dox.* ***B****. The number of rearing of experimental mice in the open field arena per bin (= 5 min) at 0 WOD, 2 WOD, 2 WOD + 2 weeks back on Dox, 2 WOD + 4 weeks back on Dox , and 2 WOD + 6 weeks back on Dox.* ***C****. The relative central distance (%) that the experimental mice travelled in the open field arena per bin (= 5 min) at 0, 2, 2 + 2, 2 + 4, and 2 + 6. Data as mean ± SD. Control (5M, 6F), rNLS8 (3M, 8F). * as p < 0.05, ** p < 0.01, *** p < 0.001 by repeated one-way ANOVA.*

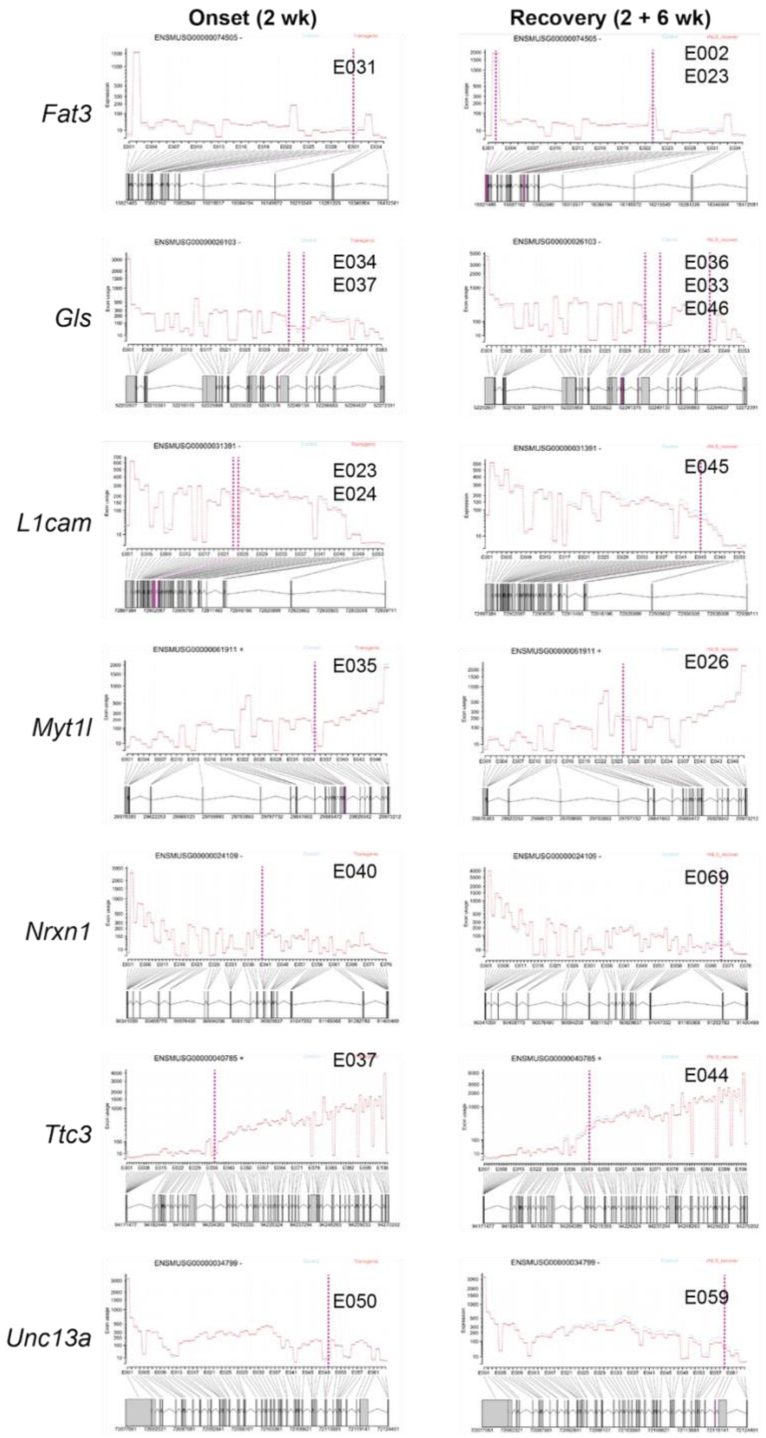

Supplementary Figure 2. Differential exon usage in representative neuronal genes associated with ALS/FTD, which were common to disease onset and recovery cortex in rNLS8 mice. *Left panels are onset (2 WOD) and right panels are recovery (2 WOD + 6 weeks back on Dox). Splicing plots showing exon usage at each exon feature.*

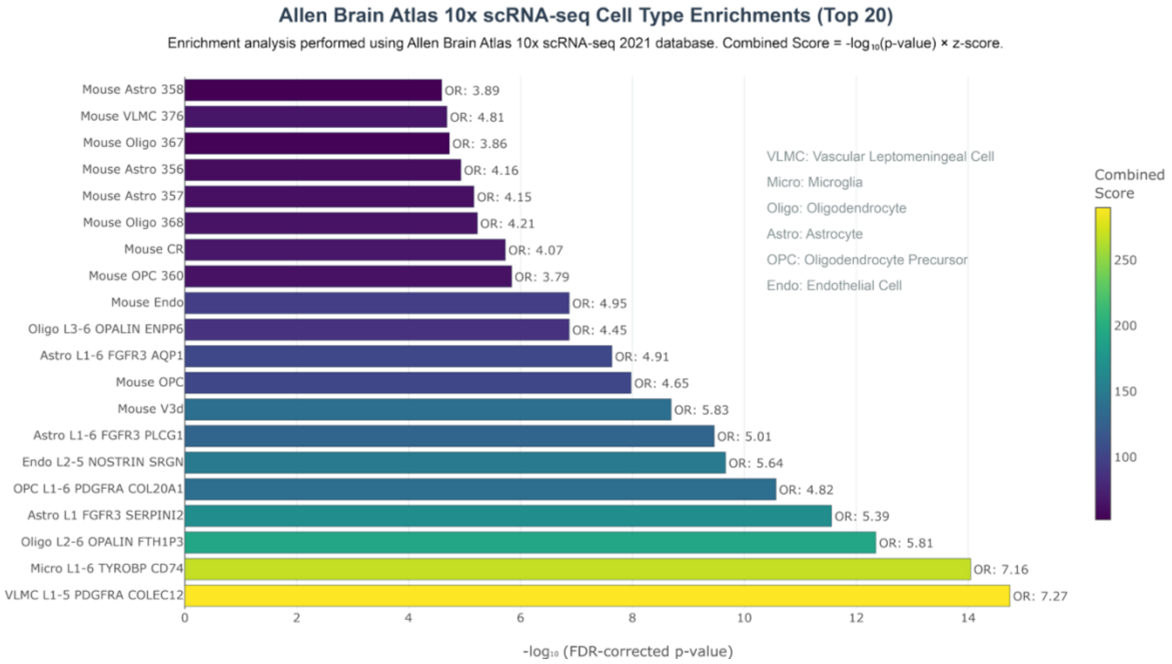

Supplementary Figure 3. Top 20 significantly enriched cell types in the DEU gene set from recovery rNLS8 cortex using the Allen Brain Atlas 10x scRNA-seq 2021 database. *Bars represent -log$_{10}$(FDR-corrected p-values) for over-representation analysis of 173 genes exhibiting alternative splicing in the rNLS8 cortex in recovery (2 week off dox and 6 weeks on dox)**.** Bar colours indicate combined enrichment scores (product of -log$_{10}$(p-value) and z-score), with yellow representing highest scores. Cell type abbreviations: VLMC, vascular leptomeningeal cells; Micro, microglia; Oligo, oligodendrocytes; Astro, astrocytes; OPC, oligodendrocyte precursor cells; Endo, endothelial cells. Odds ratios (OR) are displayed adjacent to bars. Statistical significance threshold: FDR < 0.05 using Benjamini-Hochberg correction.*

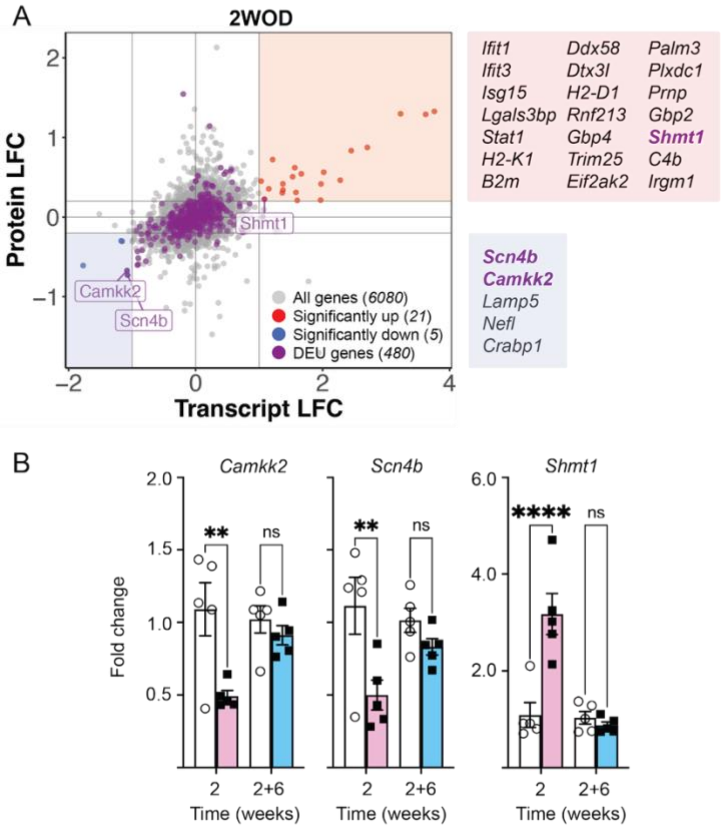

Supplemental Figure 4. Most alternatively spliced genes are not significantly altered at the protein or transcript level at disease onset in rNLS8 mice. A. *Scatter plot correlating protein and transcript abundance (log fold change; LFC) in the cortex. All genes with corresponding protein abundance data are grey and genes with significant differential exon usage (DEU) are purple. Genes with significantly increased or decreased protein*[4] *and transcripts by transcriptomic data (from this study) are red and blue, respectively, and are provided as lists ranked by their protein abundance. Scn4b and Camkk2 are genes that show DEU and have significantly decreased protein and transcript abundance. Shmt1 shows DEU and has significantly increased protein and transcript abundance.* ***B.*** *QPCR analyses confirmed the significant downregulation of Camkk2 and Scn4b and upregulation of Shmt1 at onset (2 WOD), which in each case was followed by normalisation of levels at recovery (2 WOD + 6 weeks back on Dox). N = 5. Mean ± SEM. ** as p < 0.01 and **** as p < 0.0001 by t-test.*

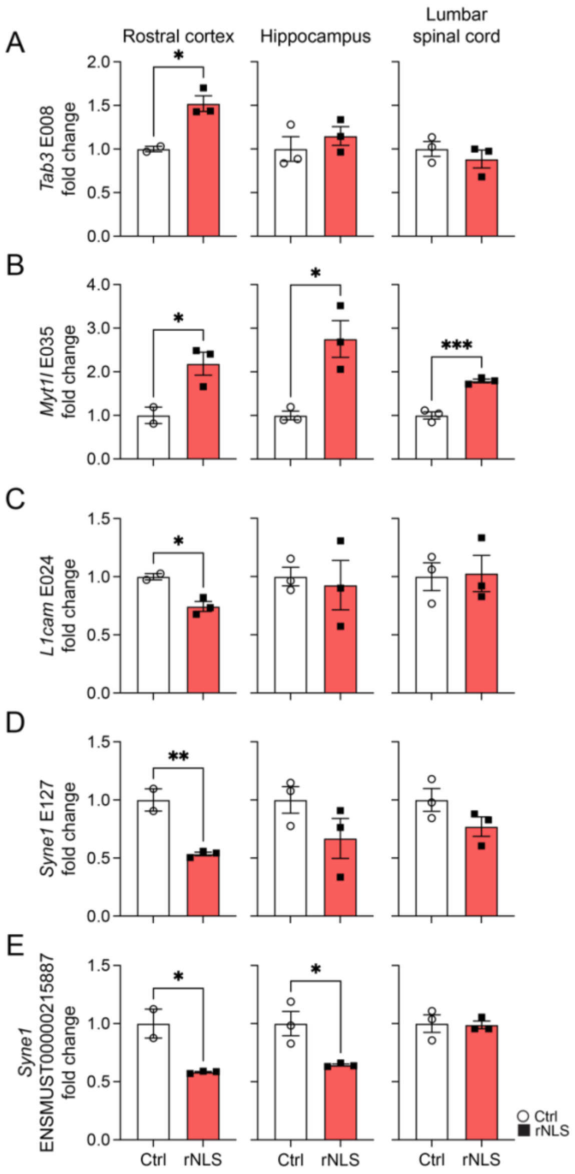

Supplemental Figure 5. DEUs in persistent alternatively spliced genes are enriched in the rostral cortex compared with other regions of the CNS. *QPCR analyses of DEUs in **(A)** Tab3 E008, **(B)** Myt1l E035, **(C)** L1cam E024, **(D)** Syne1 E127, and **(E)** Syne1 ENSMUST00000215887 in the rostral cortex, hippocampus, and lumbar spinal cord of control and rNLS8 mice at onset (2WOD). The exon event number nomenclature matches those from the output of DEXSeq, except for Syne1 ENSMUST00000215887, where primers were designed to specifically target the Syne1 isoform encompassed by E127-148. N=2-3. Mean ± SEM. * as p < 0.5 and ** as p < 0.01 by t-test.*

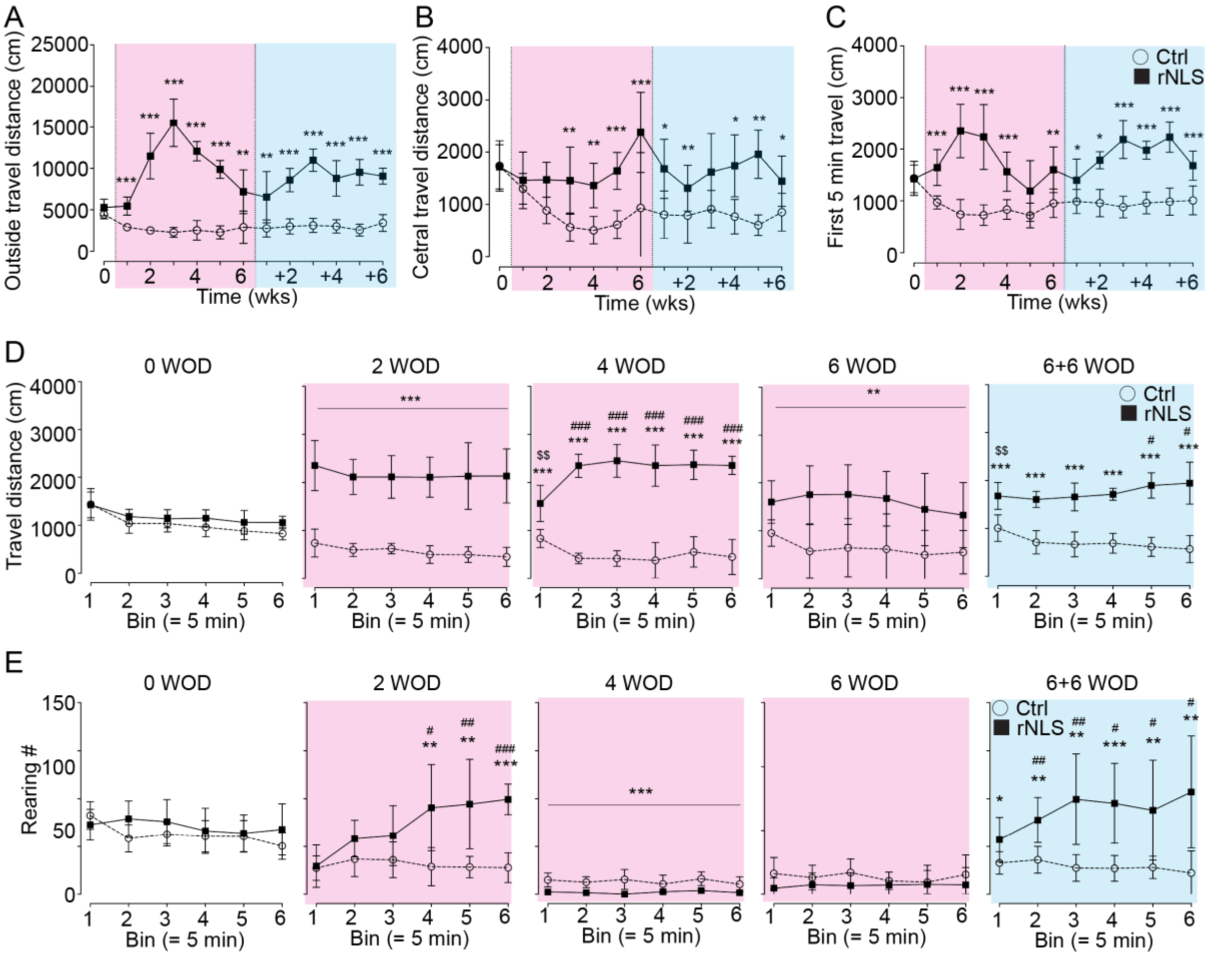

Supplementary Figure 6. rNLS8 mice display hyperlocomotion, hyperactivity phenotypes. (A) *increase outside travel distance and* ***(B)*** *inside travel distance.* ***(C)*** *Total travel distance in the initial five minutes of the open field test during disease. The temporal profile of travel distance (cm,* ***D****) and rearing number (****E****) per bin (=5 min) that the experimental mice travelled in the open field arena at timepoints as shown. Control (8F), rNLS8 (6F). Mean ± SD. * as $p < 0.05$, ** $p< 0.01$, *** $p < 0.001$, **** $p < 0.0001$ between control and rNLS8 groups by repeated t-test. $ as $p < 0.05$, $$ $p< 0.01$ between first bin (=5 min) and other bins within the control group revealed by Post-hoc tests; # as $p < 0.05$, ## $p< 0.01$, ### $p< 0.01$ between first bin (=5 min) and other bins within the rNLS8 group revealed by Post-hoc tests.*

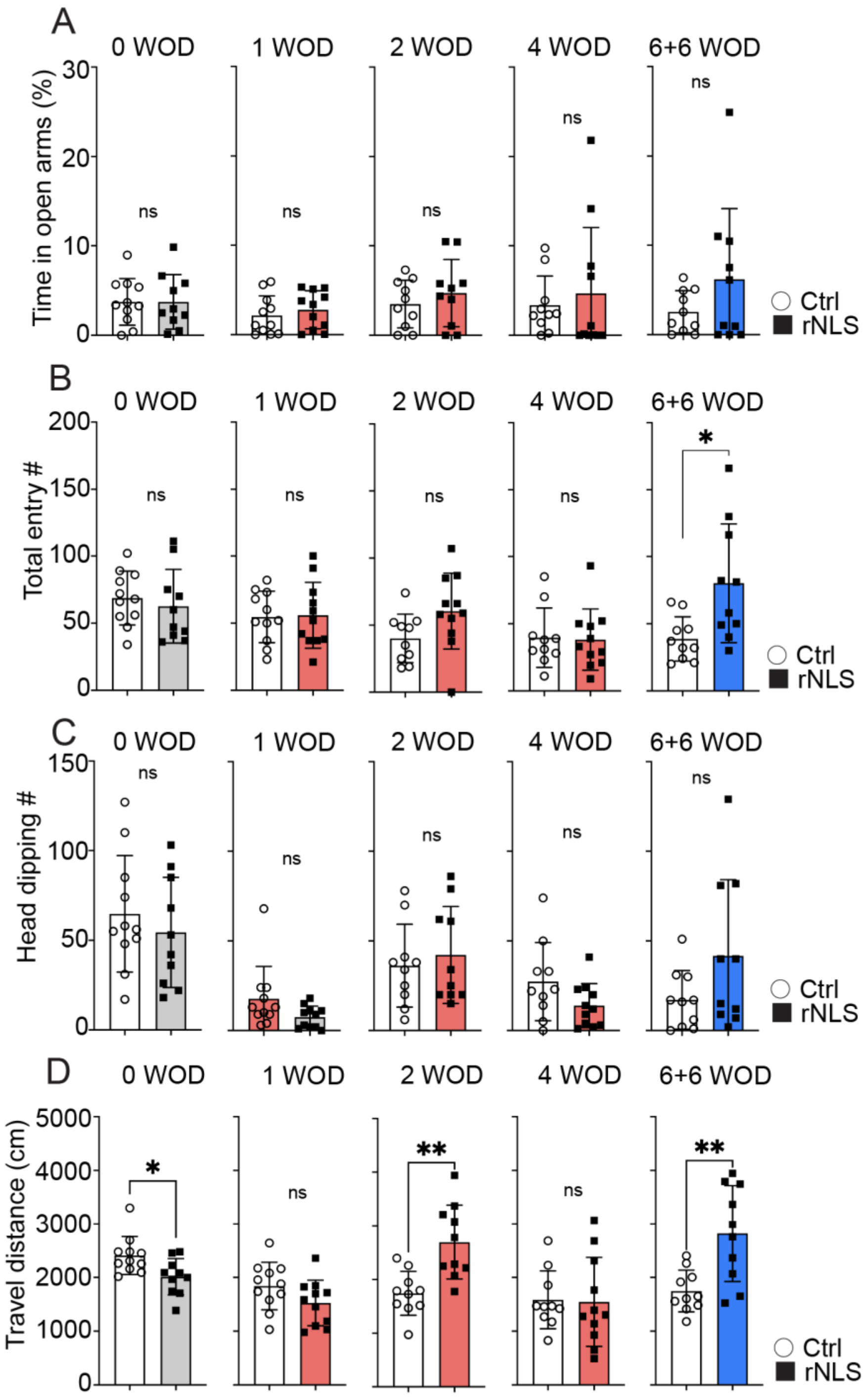

Supplementary Figure 7. rNLS8 mice exhibit no significant anxiety-like behaviours in the elevated plus maze test. (A) *relative time spent (%) in the open arms.* ***(B)*** *Head dipping number. rNLS8 mice show slightly increased total entry number* ***(C)*** *at recovery phase, and increased travel distance (cm) at disease onset and recovery phase* ***(D)****. Control (5M, 6F), rNLS8 (5M, 6F). Data as mean ± SD. * as p < 0.05, ** p < 0.01 by t-test.*

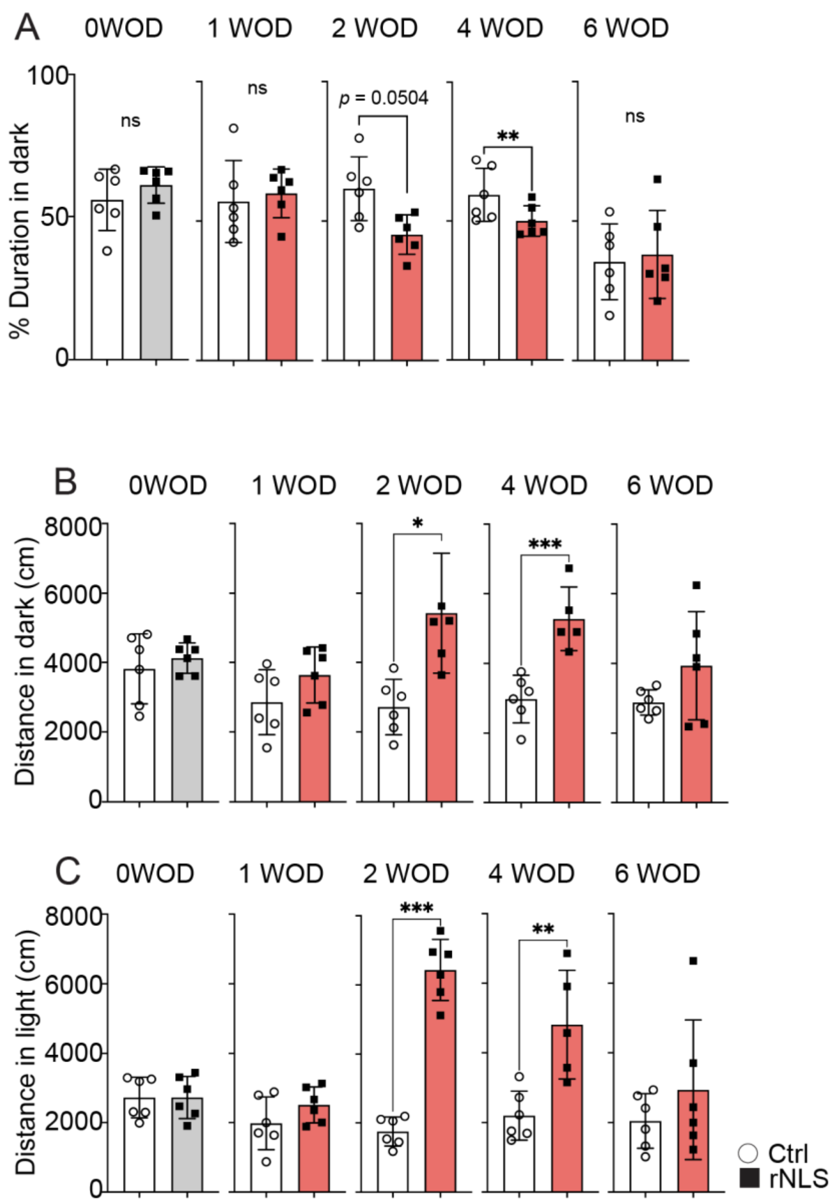

Supplementary Figure 8. rNLS8 mice exhibit signs of reduced anxiety-like behaviour related to light. (A) *The relative duration (%) and* ***(B)*** *the total travel distance (cm) in the testing chamber in* ***(B)*** *the dark area and* ***(C)*** *the light area. The light area in the open field arena divided equally by a light and dark area over 30 min at baseline before the removal of Dox (0 week off Dox), at 2, 4 and 6 weeks off Dox (WOD). Data as mean ± SD. Control (6F), rNLS8 (6F). * as p < 0.05, * p < 0.01, *** p < 0.001 by t-test.*

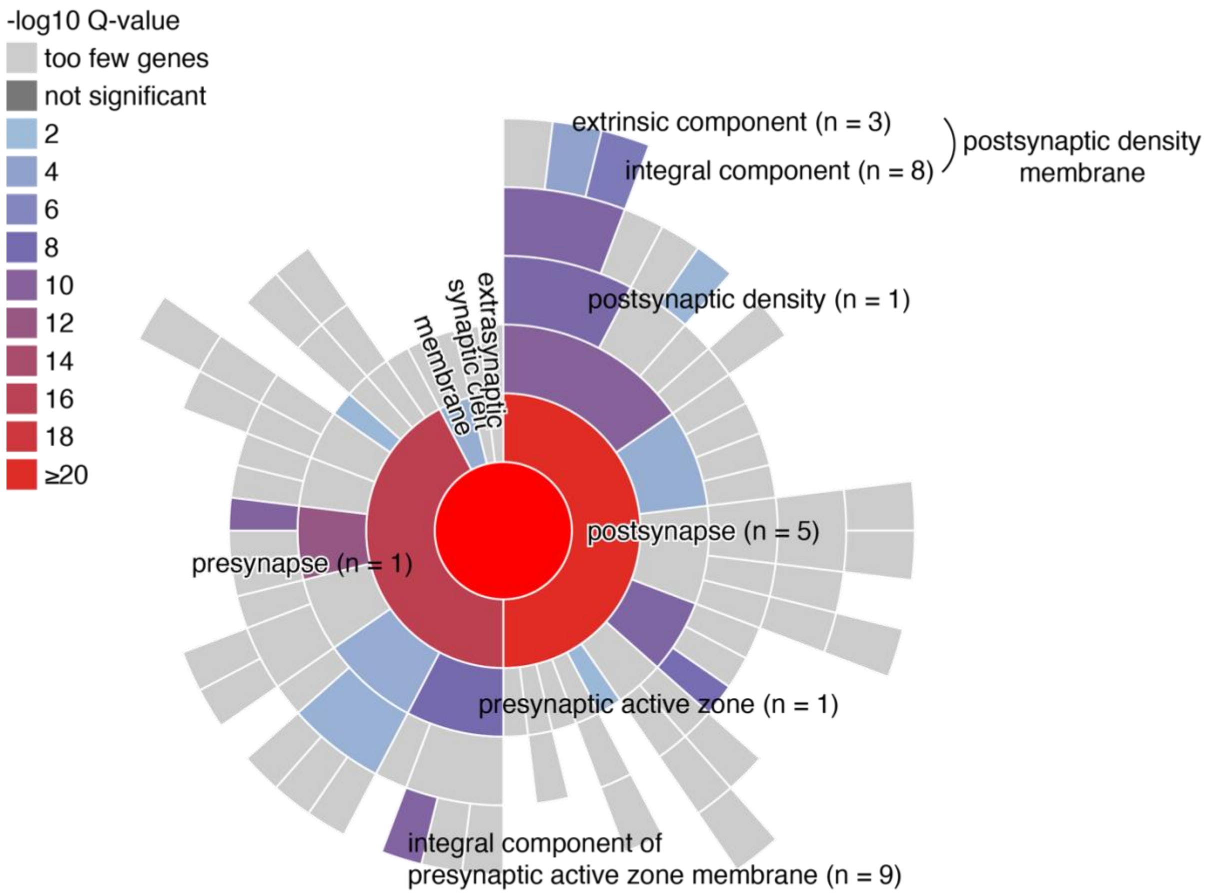

Supplementary Figure 9. Synaptic protein annotations of the glutamatergic synapse proteins that persistently significantly decreased in disease (2WOD) and recovery (2 WOD + 6 weeks on dox) by SynGO[5]. *Proteins identified to be persistently significantly decreased in rNLS8 cortex (n = 114) at 6 weeks off dox were input to the SynGO webtool showed that altered proteins span both the pre- and post-synapse.*

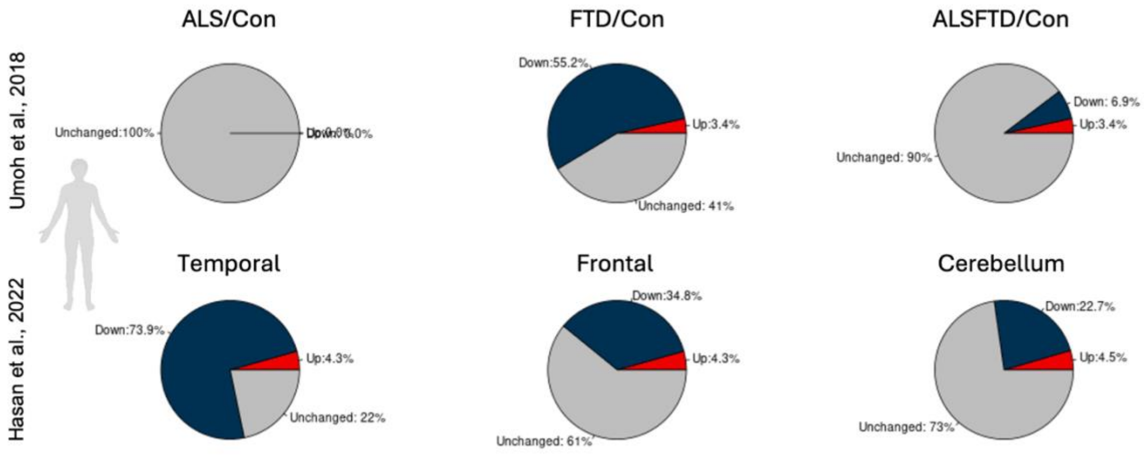

Supplementary Figure 10. Glutamatergic synapse proteins that are persistently decreased in rNLS8 late disease and recovery phase are also significantly decreased in the proteomics[6] and transcriptomics[7] datasets from human post-mortem tissue. *A subset of n = 40 persistent significantly decreased proteins, from the rNLS8 cortex "glutamatergic synapse" gene ontology term, were input into the* *TDP-map* *webtool. A total of n = 33 proteins/genes were detected in the transcriptomic[7] and proteomic[6] datasets of human post-mortem brain tissue and here, pie charts depict the direction of change (blue = down, red = up, grey = unchanged) and the % represents the proportion of proteins/genes that were up, down, or unchanged of the n = 33 present in the datasets.*

Supplementary Material References

1. Chen EY, Tan CM, Kou Y, Duan Q, Wang Z, Meirelles GV *et al.* Enrichr: interactive and collaborative HTML5 gene list enrichment analysis tool. *BMC Bioinformatics* 2013; **14**(1)**:** 128.

2. Kuleshov MV, Jones MR, Rouillard AD, Fernandez NF, Duan Q, Wang Z *et al.* Enrichr: a comprehensive gene set enrichment analysis web server 2016 update. *Nucleic Acids Research* 2016; **44**(W1)**:** W90-W97.

3. Yao Z, van Velthoven CTJ, Nguyen TN, Goldy J, Sedeno-Cortes AE, Baftizadeh F *et al.* A taxonomy of transcriptomic cell types across the isocortex and hippocampal formation. *Cell* 2021; **184**(12)**:** 3222-3241.e3226.

4. San Gil R, Pascovici D, Venturato J, Brown-Wright H, Mehta P, Madrid San Martin L *et al.* A transient protein folding response targets aggregation in the early phase of TDP-43-mediated neurodegeneration. *Nat Commun* 2024; **15**(1)**:** 1508.

5. Koopmans F, van Nierop P, Andres-Alonso M, Byrnes A, Cijsouw T, Coba MP *et al.* SynGO: An Evidence-Based, Expert-Curated Knowledge Base for the Synapse. *Neuron* 2019; **103**(2)**:** 217-234 e214.

6. Umoh ME, Dammer EB, Dai J, Duong DM, Lah JJ, Levey AI *et al.* A proteomic network approach across the ALS-FTD disease spectrum resolves clinical phenotypes and genetic vulnerability in human brain. *EMBO Mol Med* 2018; **10**(1)**:** 48-62.

7. Hasan R, Humphrey J, Bettencourt C, Newcombe J, Consortium NA, Lashley T *et al.* Transcriptomic analysis of frontotemporal lobar degeneration with TDP-43 pathology reveals cellular alterations across multiple brain regions. *Acta Neuropathol* 2022; **143**(3)**:** 383-401.
